# Targeting macrophages: a novel treatment strategy in solid tumors

**DOI:** 10.1186/s12967-022-03813-w

**Published:** 2022-12-12

**Authors:** Mengmeng Liu, Lina Liu, Yongping Song, Wei Li, Linping Xu

**Affiliations:** 1 Department of Research and Foreign Affairs, The Affiliated Cancer Hospital of Zhengzhou University and Henan Cancer Hospital, Zhengzhou, 450008 China; 2 Department of Hematology, The First Affiliated Hospital of Zhengzhou University, Zhengzhou, 450052 Henan China; 3 Academy of Medical Sciences of Zhengzhou University, Zhengzhou, 450052 China; 4 Department of Hematology, The Affiliated Cancer Hospital of Zhengzhou University and Henan Cancer Hospital, Zhengzhou, 450008 China

**Keywords:** Tumor-associated macrophages, Solid tumors, Immunotherapeutic strategies, CD47 mAb, CD47 based BsAb, CAR-M

## Abstract

In the tumor microenvironment (TME), tumor-associated macrophages (TAMs) are the most abundant immune cells, which act as a key regulator in tumorigenesis and progression. Increasing evidence have demonstrated that the TME alters the nature of macrophages to maintain dynamic tissue homeostasis, allowing TAMs to acquire the ability to stimulate angiogenesis, promote tumor metastasis and recurrence, and suppress anti-tumor immune responses. Furthermore, tumors with high TAM infiltration have poor prognoses and are resistant to treatment. In the field of solid tumor, the exploration of tumor-promoting mechanisms of TAMs has attracted much attention and targeting TAMs has emerged as a promising immunotherapeutic strategy. Currently, the most common therapeutic options for targeting TAMs are as follows: the deletion of TAMs, the inhibition of TAMs recruitment, the release of phagocytosis by TAMs, and the reprogramming of macrophages to remodel their anti-tumor capacity. Promisingly, the study of chimeric antigen receptor macrophages (CAR-Ms) may provide even greater benefit for patients with solid tumors. In this review, we discuss how TAMs promote the progression of solid tumors as well as summarize emerging immunotherapeutic strategies that targeting macrophages.

## Introduction

**Fig. 1:**
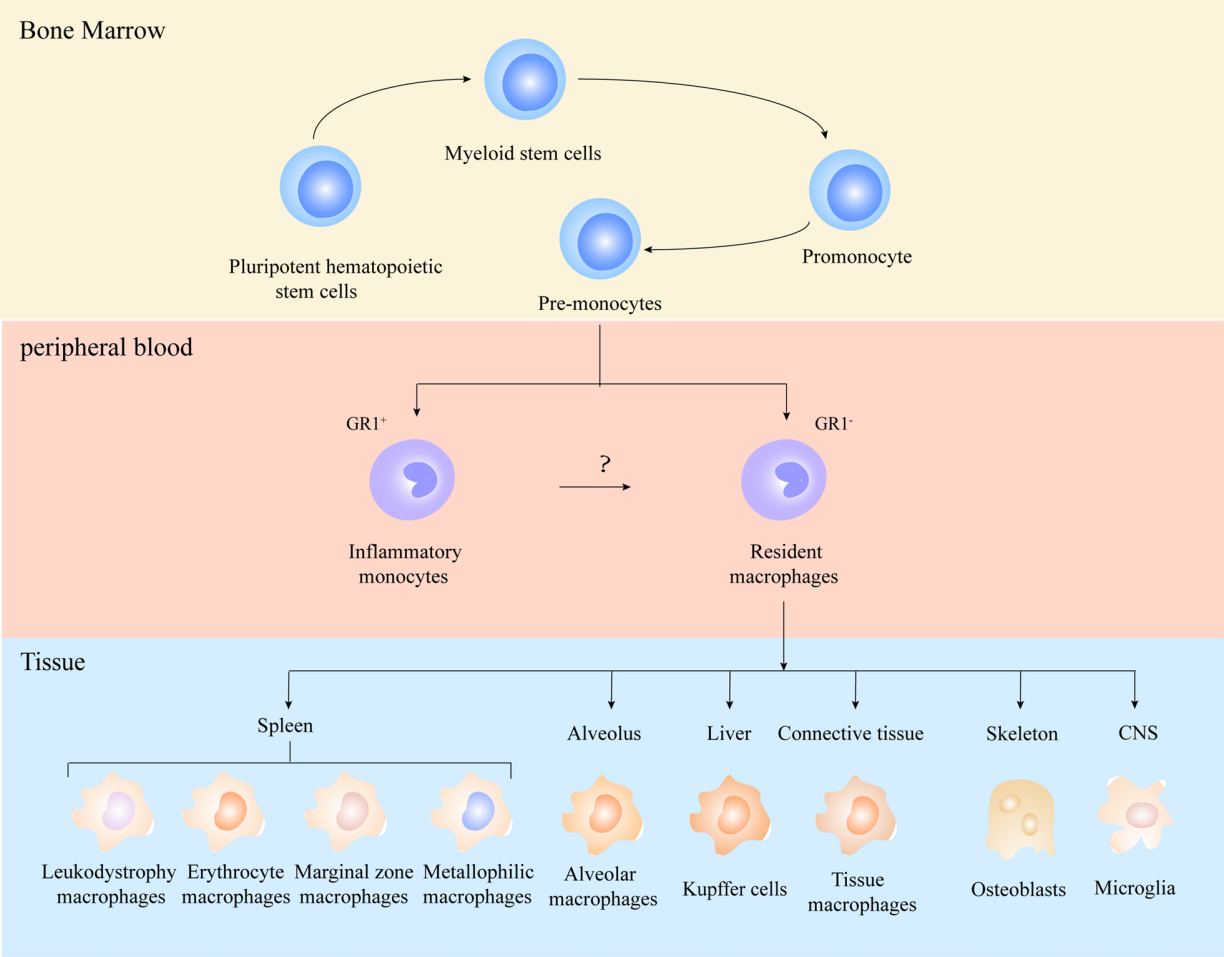
The origin of macrophages. Macrophages originate from pluripotent hematopoietic stem cells in the bone marrow, where they differentiate into promonocytes and enter the peripheral blood. In the peripheral blood, pro-monocytes differentiate into Gr1^+^ inflammatory monocytes and Gr1^−^ macrophages. Macrophages located in different tissues are called resident macrophages, such as leukodystrophy macrophages, Erythrocyte macrophages, Marginal zone macrophages, Metallophilic macrophages in the spleen, alveolar macrophages in the alveolus, Kupffer cells in the liver, tissue macrophages in the connective tissue, osteoblasts in the bone, and microglia in the central nervous system

Tumor microenvironment (TME) consists of peripheral blood vessels, stromal cells, endothelial cells, tumor-associated fibroblasts, and immune cells [1–3]. There are several types of immune cells in the TME, including T cells, natural killer (NK) cells, dendritic cells (DCs), neutrophils, macrophages, and myeloid-derived suppressor cells (MDSCs) [4–6]. Significantly, tumor-associated macrophage (TAM) is the largest immune cell population in the TME [7]. Macrophages originate from bone marrow (BM) hematopoietic stem cells and develop into pre-monocytes and monocytes in the BM microenvironment. Monocytes enter the bloodstream from the BM microenvironment and cross the blood vessels into tissues and organs, mature into macrophages, and are found in tissues and organs. Examples include microglia in the brain, osteoblasts and erythroblastic island macrophages in BM, hepatic macrophages in the liver, red pup macrophages in spleen, and alveolar macrophages in the lungs [7–11]. A critical component of the intrinsic immune system, macrophages are the body’s first defense against pathogen invasion and activate the adaptive immune system (Fig. 1). Therefore, macrophages may be a promising target in many human diseases, including cancer immunotherapy.

**Fig. 2:**
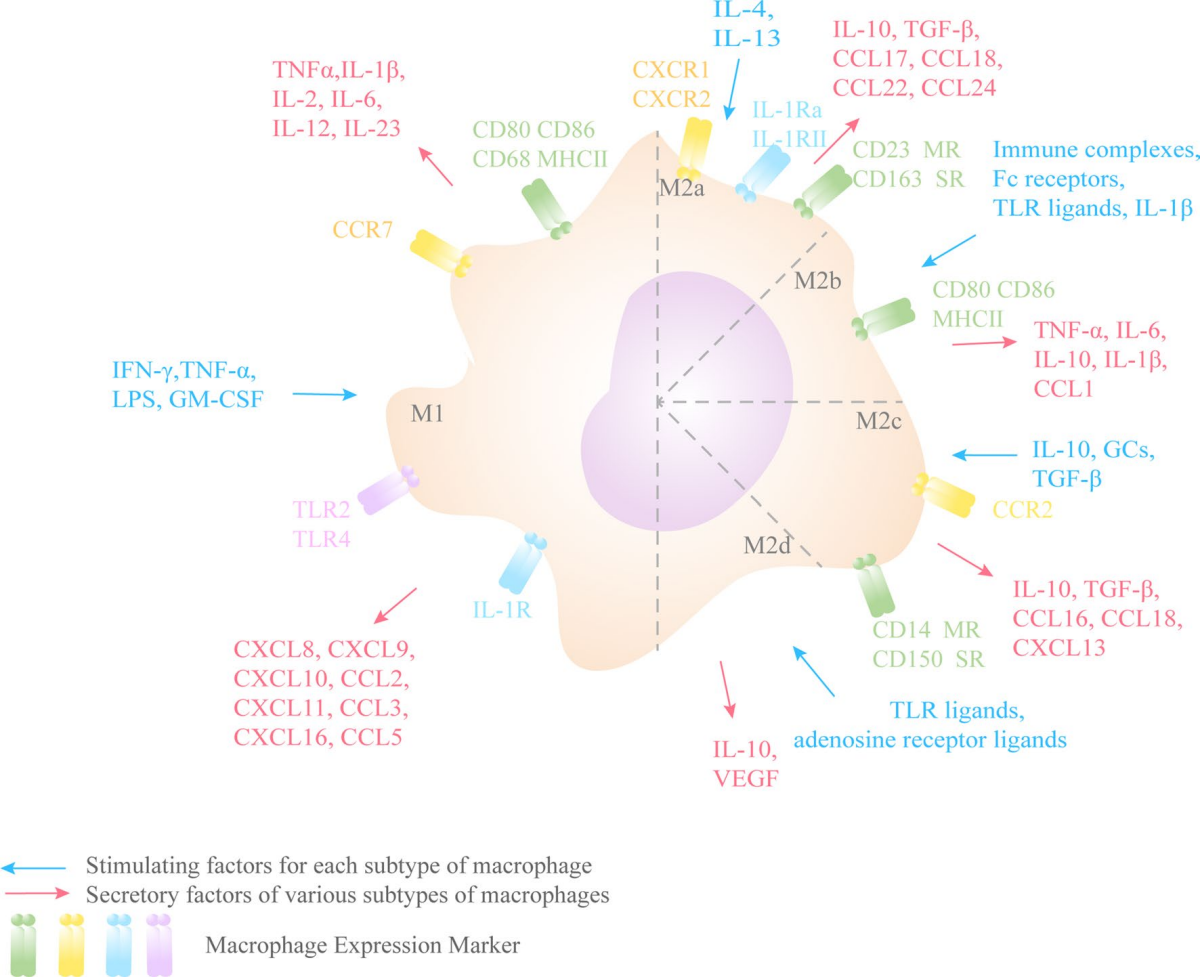
Different stimulating factors can polarize macrophages toward different subtypes. IFN-γ, TNF-α, LPS, and GM-CSF can induce macrophages to polarize toward the M1 phenotype, resulting in high expression of pro-inflammatory cytokines such as IL-12 and low expression of IL-10. IL-4, IL-10, IL-13, TGF-β, and GCs stimulate macrophages to polarize toward M2, resulting in high expression of IL-10 and low expression of IL-12. Different stimulating factors induce different M2 isoforms, causing them to exhibit different functions

Due to the strong plasticity and heterogeneity of macrophages, TAMs can exhibit different phenotypes and characteristics depending on the cytokines, pathogen-associated molecular patterns, metabolic signals, cell–cell interactions, and tissue-specific signals in the TME [12]. According to the different phenotypic and functional characteristics of macrophages and their roles in response to Th1 and Th2, they can be divided into classically activated M1-type and alternatively activated M2-type macrophages (Fig. 2) [13]. M1 macrophages are usually induced by cytokines secreted by Th1 cells (IFN-γ and tumor necrosis factor α, TNF-α) or bacterial lipopolysaccharides (LPS). These macrophages can secrete higher levels of proinflammatory cytokines, such as TNF-α and IL-1β, IL-2, IL-6, IL-12, and IL-23, while IL-10 is secreted at lower levels. M1 macrophages are involved in killing pathogens and tumor cells as inducible and effector cells in the Th1-type immune response. Thus, M1 macrophages have a tumor-suppressive effect [14, 15]. M2 macrophages activate STAT6 via IL-4Rα and are polarized by the Th2 cytokines IL-4 and IL-13. In addition to IL-4 and IL-13, cytokines such as IL-10 can also regulate M2 macrophage polarization by activating STAT3 through IL-10R [16]. M2 macrophages are characterized by the secretion of anti-inflammatory cytokines, namely the low expression of IL-12 and high production of IL-10 and transforming growth factor-β (TGF-β) [17, 18]. The main function of M2 macrophages is the trophic effect on tissues, while their weak antigen-presenting ability inhibits inflammatory responses and promotes wound healing, angiogenesis and tissue repair [14, 19]. Depending on the activating stimulus, M2 macrophages can be further divided into four subpopulations, namely M2a, M2b, M2c, and M2d [20]. M2a is activated by IL-4 and IL-13 induction and can cause Th2-type immune responses, type II inflammatory responses, allergic responses, and parasite killing and sequestration responses. The activation triggered by Fc receptors and immune complexes is called M2b, which expresses high IL-10 and low IL-12, and also secretes TNF, IL-1, and IL-6, thus promoting the activation of Th2 and participating in immune regulatory responses. M2c is induced and activated by IL-10 and glucocorticoid (GCs), which promotes the processes of matrix deposition and fibrotic tissue repair and reconstruction. Finally, TLR agonists induce a fourth M2-type macrophage called M2d through adenosine receptor agonists [21, 22]. M2-type macrophages are closely associated with negative immune regulation and immune tolerance. And in the TME, M2 macrophages inhibit inflammatory responses, promote tissue remodeling and angiogenesis, and thus have a function in promoting tumor progression [23].

TAMs are a heterogeneous group of cells differentiated from BM-derived circulating monocytes with different genomic and functional characteristics, exhibiting the dual function of promoting and resisting tumor progression in their interaction with tumor cells [22, 24, 25]. The current study reveals that the nature of TAMs change with the continuous alteration of spatial availability and growth factors in the TME [26]. During tumor progression, TAMs are gradually transformed from an anti-tumor phenotype at tumor initiation into part of an anti-inflammatory circuit that promotes tumor progression and plays a crucial regulatory role in all aspects of solid tumor progression [27]. Furthermore, TAMs are subtypes of M2 macrophages that are closely associated with tumor progression [28, 29]. Previously, TAMs were thought to inhibit or promote tumor progression by affecting cytokines, leukocytes, and inflammatory mediators. However, many clinical and preclinical studies have demonstrated the complexity of the interaction between TAMs and tumor cells [25]. As research continues in this field, there is increasing evidence that TAMs can interact with other immune cells in the TME at various stages of tumor progression, thereby promoting tumor cell initiation and the formation of an immunosuppressive microenvironment, stimulating tumor angiogenesis, and enhancing tumor cell proliferation and metastasis, ultimately accelerating tumor progression [15] (Table 1). As the interrelationship between TAMs and malignant tumors has become clearer, TAMs have emerged as potential biomarkers and therapeutic targets for the diagnosis and prognosis of various solid tumors.

**Table 1:** Signals involved in the interaction of TAMs with tumor cells

Signaling pathways	Modulation of TAMs	Regulation of tumor cells	Functions
WNT signaling pathway	Wnt signaling-inducible proteins released from tumor cells mediate TAMs recruitment and m2-like polarization	Wnt proteins released from TAMs augment the stemness of tumor cells through a β-catenin-dependent pathway and promote the invasiveness of tumor cells through a β-catenin-independent pathway	Facilitates tumor advancement through β-catenin-dependent and β-catenin-independent pathways; Wnt5a promotes tumor cell invasion through an atypical pathway
PI3K signaling pathway	Various cytokines, including EGFR, activate the PI3K pathway and contribute to the recruitment of TAMs and their polarization to the M2 phenotype, providing a suitable environment for tumor progression	Tumor cells release succinate via the pi3k-hypoxia inducible factor 1a (HIF1A) axis, thus promoting macrophage transformation	Enhance the proliferation, differentiation and migration of tumor cells
STAT3 signaling pathway	Activation of JAK3/STAT3 pathway and secretion of IL-6 and IL-10 induce TAMs to M2 polarization	IL-6 secreted by TAMs activates the JAK2/STAT3 pathway, which stimulates tumor cell invasion and metastasis	Enhancement of tumor proliferation, invasion and metastasis
NF-kB signaling pathway	Decreased phosphorylation of RELA protein stimulates TAMs to M2 polarization	Cytokines secreted by M2 enhance TNF and iNOS expression in tumor cells, resulting in activation of the NF-kB pathway	Inhibition of apoptosis of tumor cells and promotion of tumor angiogenesis and metastasis
Exosome signaling	Exosomes secreted by TAMs promote tumor neoangiogenesis and tumor cell invasiveness	Tumor cell-derived exosomes promote tumor progression by activating TAMs	Mediates communication between tumor cells and TAMs to create conditions for tumor cell survival and development

Currently, research is focused on the mechanism of action of TAMs in TME, how to regulate their function, and how to use them rationally to treat tumors, all with the aim of improving the effectiveness of various treatment options. In this review, we focus on the mechanisms through which TAMs promote solid tumor progression and summarize emerging immunotherapeutic strategies based on TAMs.

## Mechanisms of tumor progression mediated by macrophages

### Promotion of angiogenesis

The blood supply requirements of tumors are usually the rate-limiting step in tumor progression. Malignant tumors grow rapidly and tumor progression requires a network of blood vessels that deliver oxygen and nutrients as well as dispose of metabolic waste [30, 31]. The environment in which tumor cells are located is in a state of hypoxia and increased acidity. Hypoxia is the main driver of tumor angiogenesis, leading to an imbalance between proangiogenic and antiangiogenic factors through the secretion of numerous angiogenic factors [32, 33]. This stimulates the proliferation and wandering activity of vascular endothelial cells, leading to haphazard angiogenesis and inducing the formation of new blood vessels. Tumor vasculature is usually abnormal, immature, and leaky compared with normal vasculature. This onset of angiogenesis is called the “angiogenic switch” and it occurs at different stages of tumor progression. [34]

TAMs accumulate in peritumor vessels and are major players in the angiogenesis process of solid tumors. The number of TAMs was found to be significantly higher around proliferating vessels than around normal tissue vessels [35]. Moreover, a study demonstrated that the massive depletion of TAMs or knockdown of the vascular endothelial growth factor (VEGF) gene in macrophages delays the angiogenic transition process, whereas restoration of this gene expression restores the angiogenic function of macrophages [36].

In addition, TAMs secrete a variety of proangiogenic factors, such as VEGF, TNF-α, IL-1β, IL-6, IL-8, and various chemokines, including CXCL3, 4, 8, 9, and 10; and CCL2-5 [15, 37, 38]. The VEGF family has three related tyrosine kinase receptors, namely VEGFR1, VEGFR2, and VEGFR3. VEGFR2 plays a crucial role in angiogenesis through activating the MAPK and PI3K signaling pathways, which in turn activate downstream ERK1/2 or mTOR ligands, leading to tumor growth and angiogenesis. In addition, different types of T cells can regulate the function of TAMs and influence their role in angiogenesis. CD8^+^ cytotoxic T cells and CD4^+^ Th1 cells produce IFN-γ, inhibit endothelial cell proliferation, and induce the production of the vasopressor chemokines CXCL9, 10, and 11 in TAMs; in turn, this activates downstream ERK1/2 or mTOR ligands, leading to tumor growth and angiogenesis [39]. Moreover, regulatory T cells (Tregs) suppress the production of INF-γ-expressing CD4^+^ Th1 cells and secrete VEGF via hypoxia-induced CCL28 [40].

Hypoxia-inducible factor (HIF-1α) expressed by TAMs is also important in promoting angiogenesis [25]. HIF-1α upregulates NF-kB expression and leads to the recruitment of monocytes and TAMs, as well as to the M2 phenotypic polarization of TAMs, thus promoting tumor recurrence and metastasis [41]. Endothelial progenitor cells can synthesize mature endothelial cells from scratch in the presence of TAMs. IL-6 released by TAMs activates Janus kinase/signal transducers and JAK-STAT signaling pathways in recruited endothelial progenitor cells, facilitating the generation of vascular endothelial cells from endothelial progenitor cells [35]. IL-10 stimulates TAMs to activate STAT3 signaling, which promotes the release of VEGF-A and supports tumor angiogenesis [42]. In addition, other factors such as WNT7B,TGF-β, and thymidine phosphorylase further support the generation of the vascular network in the TME by recruiting and activating other cells, such as endothelial cells and fibroblasts [43].

Studies on malignancies have focused on the secretion of soluble signaling molecules, such as cytokines and chemokines. Recently, studies have reported exosomes that regulate the exchange of TME substances and information [44]. Exosomes are small cellular vesicles that originate from cells that carry genetic information (e.g., proteins and nucleic acids). They are capable of regulating intercellular information transfer and material exchange to influence the function of target cells. Different exosomes are involved in different stages of cancer cell survival, growth, and metastasis in malignant tumors [45, 46]. M2 macrophage-derived exosomes are associated with the promotion of tumor angiogenesis. In pancreatic ductal adenocarcinoma (PDAC), the exosomes secreted from M2 macrophages carry miR-155-5p and miR-221–5 and act as carriers that transport miRNAs to endothelial cells when they detach from cells [47]. The transported miR-155-5p and miR-221–5 bind to E2F2 in endothelial cells, a gene that inhibits endothelial cell angiogenesis and promotes angiogenesis in PDAC, resulting in a positive correlation between M2 macrophages and the vascular density of tumor tissue [47]. Moreover, in ovarian cancer, HIF induces the release of exosomes enriched in various miRNAs, such as miR-21-3p, miR-125b-5p, and miR-181d-5p. These exosomes induce the polarization of undifferentiated macrophages toward M2 phenotype via the suppressor of cytokine signaling (SOCS) 4, 5, and STAT3 pathways [48]. In conclusion, TAMs are responsible for driving tumor angiogenesis and the associated progression of tumors, which provides a theoretical basis for an anti-angiogenesis therapy strategy that targets TAMs [33].

### Resistance to treatment

Resistance to anti-cancer treatment may be an inherent ability of cancer cells, but it is usually caused by the nonmalignant cells that constitute the TME [43]. The inability of tumor cells to recruit BM intrinsic effector cells as a result of immunotherapy is the main mechanism responsible for their immune escape and acquired resistance [49]. The current focus of cancer therapy is on discovering the causes of resistance to tumor treatments.

Several studies have been reported that TAMs are involved in the development of chemoresistance in tumor cells [33, 50–52]. Chemoresistance includes intrinsic resistance or acquired resistance induced by multiple factors, such as drug inactivation, excessive drug efflux, and alterations in target cells [53]. Study indicated that TAMs protect breast cancer cells from cell death caused by chemotherapeutic drug attacks with paclitaxel, etoposide, and doxorubicin [54]. Meanwhile, the infiltration of TAMs is associated with resistance to chemotherapy in colorectal cancer (CRC), and TAM-derived IL-6 inhibits the expression of tumor suppressors by activating the IL6R/STAT3 pathway, thereby inducing drug resistance in cancer cells [55]. TAMs are activated during 5-FU treatment of CRC to protect CRC cells from 5-FU chemotherapy by secreting cytokines that attenuate JNK-caspase-3 pathway-mediated apoptosis [56]. TAMs assist in the development of chemoresistance in cancer cells through various mechanisms, among which exosomes are also important in the formation of this process. In a study of PDAC resistance to gemcitabine, the authors found that TAMs secreted exosomal vesicles that were selectively internalized by tumor cells. This established a communication bridge between TAMs and tumor cells by transferring miR-365, significantly reducing the sensitivity of PDAC cells to gemcitabine [57].

Radiotherapy is also a main treatment strategy for solid tumors. The CSF-1R inhibitor was confirmed to inhibit myeloid monocyte differentiation into TAMs, and improve treatment response for glioblastoma after ionizing radiation [58, 59]. Additionally, other studies have shown that reprogramming TAMs can enhance radiotherapy effectiveness [60–62].

In addition, TAMs can lead to primary and secondary resistance of tumor cells to immune checkpoint inhibitors (ICIs) [63]. Valeria Quaranta et al. demonstrated that macrophage-derived granulin drives resistance to ICIs in metastatic PDAC [64]. In this study, they found that macrophage-derived granulin contributes to cytotoxic CD8^+^ T-cell exhaustion in metastatic PDAC and granulin-depleted tumors treated with PD-1 blockade gained anti-tumor immunity and had dramatically reduced metastatic tumor burdens [64]. Molgora et al. reported that TREM2 expression by macrophages promote tumor growth and inhibit anti-tumor immune responses. Targeting TREM2 remodels the tumor myeloid landscape to reduce the tumor growth. Significantly, it enhances anti-PD-1 immunotherapy in animal models of solid tumors [65].

### Promotion of tumor recurrence and metastasis

The instability of the tumor genome and the loss of normal cellular regulatory processes can lead to the expression of tumor antigens that distinguish tumor cells from normal cells and are thus recognized and cleared by the immune system. This process is known as tumor immunosurveillance [66]. The pressure exerted by the immune system on the tumor gradually decreases as the tumor progresses, and the tumor cells develop immunosuppressive and tolerant mechanisms to avoid clearance by the immune system [67]. These mechanisms eventually allow the immune system to progress from suppressing the tumor to shaping it. As a result, tumor cells are capable of metastasizing away from the primary lesion. Tumor metastasis is a feature of all malignancies and an important factor causing tumor-related death [27]. The process of tumor metastasis is multi-stage. Metastasis begins with the detachment of tumor cells from the primary tumor site and then transports through multiple pathways in the body. Eventually, it reaches tissues that are not contiguous with the primary site to colonize and continue to grow to form secondary tumors with the same pathological nature as the primary tumor [68]. The occurrence of metastasis is influenced not only by the driving force of the tumor itself but also by the environment surrounding the tumor cells. In the TME, TAMs are closely associated with tumor metastasis [69].

TAMs secrete signaling factors that signal metastasis and survival to tumor cells and suppress cytotoxic T cells to promote metastasis. TAMs exert pro-tumor and immunosuppressive functions by secreting IL-10 and TGF-β, VEGF, expressing PD-1, and depleting arginine to suppress T cells’ anti-tumor function [27]. Thus, an aggressive TME is formed that enhances the invasive ability of tumor cells. The EGF family ligands secreted by macrophages and CSF-1 secreted by tumor cells form a paracrine loop between macrophages and tumor cells. Studies have illustrated that the invasive TME formed by TAMs helps tumor cells to pass directly through this loop, actively participating in the process of tumor metastasis and promoting the spread of metastatic cancer cells [70, 71]. The positive feedback loop formed by Granulocyte–macrophage colony-stimulating factor (GM-CSF)-CCL18 facilitates cancer cells to maintain or promote their mesenchymal phenotype, creating a positive condition for cancer cell metastasis. CCL18, one of the major cytokines released by TAMs, enhances cancer cell metastasis by activating the NF-kB signaling pathway in breast cancer metastasis. Furthermore, inflammatory cytokines such as GM-CSF, CCL2, IL-8, and growth-related oncogene (GRO) can be induced to NF-kB target genes by CCL18. These increased cytokines are critical in the TME; GM-CSF is the cytokine responsible for macrophage activation, CCL2 can recruit monocytes, GRO mainly recruits neutrophils, and IL-8 mainly promotes angiogenesis. Therefore, the GM-CSF-CCL18 loop may be a potential therapeutic target for cancer metastasis [72]. Nevertheless, GM-CSF-induced erythrocytopenia is also noteworthy [73].

In preclinical cancer model studies of breast cancer, the upregulation of CCL2 expressed by tumor cells has been found to promote the recruitment of TAMs; moreover, VEGF-C and VEGF-D secreted by TAMs have been found to promote the lymphatic metastasis of tumor cells. Targeting CCL2 has been demonstrated to be effective, and targeting the CCL2/CCR2 signaling pathway reprograms immune angiogenesis and the TME, resulting in elevated CD8^+^ T cells, decreased M2 macrophages, reduced angiogenesis, and the enhanced effectiveness of targeted therapy and immunotherapy [74]. In addition, at the site of cervical lesions, VEGF-C, VEGF-D, and its receptor VEGFR-3 positively correlate with lesion grading by supporting the formation of lymphatic vessels [25, 75].

S100A9, also known as MRP14, is a Ca^2+^ binding protein of the S100 family. It binds to S100A8 as a homodimer or heterodimer, forming a homo-or heterodimeric complex essential for its biological activity (S100A8/A9); together, they are known as calprotective proteins. These proteins stimulate chemotaxis, cell migration, and adhesion, and they also have anti-inflammatory effects in the scavenging of oxidants, tissue repair, and elimination of inflammation [76]. S100A8 and S100A9 are low-molecular-weight intracellular calcium-binding proteins with tissue- and cell-specific expression properties. MDSCs were found to retain S100A9 expression, whereas monocyte-derived or TR-derived macrophages were mostly found to be negative [77]. Overexpression of S100A9 in naïve macrophages is sufficient to convert these cells into suppressor macrophages and polarize them toward the M2 phenotype. In studies of malignant solid tumors, such as CRC, breast cancer, and prostate cancer, S100A8 and S100A9 levels have been found to be elevated in tumor tissues compared with normal and benign tissues, and their increased expression has been associated with tumor aggressiveness and metastasis [78–80]. Their expression recruits myeloid cells and MDSCs, promotes the formation of a premetastatic microenvironment, and stimulates tumor growth and metastasis [24, 76].

In addition, WNT7B is one of the key factors secreted by TAMs to promote tumor progression and metastasis, and it promotes tumor cell invasion by affecting angiogenesis [81]. TAMs also secrete a variety of proteases, including matrix metalloproteinases (MMPs), fibrinolytic enzymes, urokinase fibrinogen activator (uPA), and serine or cysteine proteases [82]. These proteases destabilize the vascular system, increase vascular intravasation, and assist tumor cells in entering the circulatory system [43, 83, 84].

Recent studies have demonstrated that cancer induces immune stress, and TAMs are one of the immune stress products with protumor activity exported by the myeloid lineage [85, 86]. Heme oxygenase-1 (HO-1) gives TAMs the ability to break down heme at a high rate and plays a crucial role in the formation of a premetastatic TME that favors immunosuppression, angiogenesis, and epithelial-to-mesenchymal transition (EMT) [87]. This study found that tumors use the HO-1 activity of TAMs to promote immunosuppression, angiogenesis, and EMT, thereby promoting metastasis. It also demonstrated that pharmacological inhibition or myeloid-specific ablation of HO-1 attenuates the occurrence of protumor events and enhances the effectiveness of specific anti-tumor immune responses and anti-PD-1-mediated immunotherapy. Similar antitumor effects were obtained by inhibiting the recruitment of HO-1^+^ TAMs (i.e., M-CSF or C3a). This suggests that HO-1^+^ TAMs may be a target for anti-tumor therapy. [86]

### Formation of an immunosuppressive microenvironment

The progression and metabolic capacity of solid tumors are strongly influenced by various cells in the TME. The ability of tumor cells to evade immune surveillance and to grow and proliferate in the TME is the result of the interaction between tumor cells and the TME. Many reasons exist for the loss of immunogenicity of the body against tumor cells, but the main drivers are the recruitment of immunosuppressive cells and the production of immunosuppressive factors [88]. Among them, TAMs are the predominant immunosuppressive cells in the TME, suppressing the immune response by secreting cytokines and chemokines.

IL-10 secretion represents the M2-type polarization of TAMs and its inhibitory role in the anti-tumor immune response. High infiltrations of IL-10^+^ TAMs cause immune effector cells such as CD8^+^ T cells and NK cells to be fatigued or dysfunctional, depriving them of their active role in anti-tumor immune responses. IL-10 also suppresses cytotoxic T cell responses by inducing monocytes to express the co-stimulatory molecule PD-L1 and upregulating immune checkpoints, such as CTLA-4, TIM-3, and LAG-3 [89, 90]. Serum IL-10 levels have been found to positively correlate with tumor progression; moreover, studies have revealed that IL-10^+^ TAMs act as a signaling molecule that promotes immune evasion in breast, gastric, and bladder cancers, negatively correlating with overall patient survival and recurrence-free survival [90–92]. In addition, Toll-like receptor 4 (TLR4) stimulates IL-10 secretion through M2 macrophages [93], and therefore, TLR4 may also be a key signal that promotes alterations in the TME.

CCL22 is a chemokine that regulates Treg, and TAM-derived CCL22 promotes Treg recruitment at tumor sites, which inhibits cytotoxic T cell responses [94]. Moreover, in a cervical cancer studies, CCL22 in the TME induced macrophage polarization toward the M2a phenotype [95]. An increasing number of studies have demonstrated that CCL22 is a protumor chemokine and that the high infiltration of CCL22 in the TME facilitates the formation of an immunosuppressive TME.

Macrophage receptor with collagenous structure (MARCO) is a scavenger receptor expressed by macrophages, and in solid tumors, macrophages expressing MARCO represent a subpopulation of anti-inflammatory and protumor macrophages [7, 33]. In non-small-cell lung cancer (NSCLC), cancer cells drive macrophages to polarize toward the M2 phenotype to express MARCO and acquire an immunosuppressive phenotype through the release of IL-37. TAMs expressing MARCO blocked the activation of cytotoxic T cells and NK cells, inhibiting their proliferation, cytokine production, and ability to kill tumor cells. Mechanistically, MARCO^+^ macrophages enhance the proliferative activity of Tregs and IL-10 production as well as reduce CD8^+^ T cell activity, creating a suppressive microenvironment suitable for tumor cell survival [96, 97]. In a mouse model of melanoma, treatment with anti-MARCO reversed the inhibitory effect of TAMs on NK cells and synergized with T cell immunotherapy to enhance the treatment efficacy of melanoma. Similarly, experiments revealed that anti-Human MARCO antibody reactivated NK cell-mediated melanoma killing [98].

GM-CSF stimulates the proliferation and survival of macrophages, neutrophils, DCs, and microglia. In mouse disease models and in the human circulatory system, low levels of GM-CSF exert anti-tumor effects by activating DCs within tumors [72]. By contrast, at advanced stages of tumor progression, high levels of GM-CSF mainly exhibit protumor activity to reinforce the immunosuppressive microenvironment formed by M2 TAMs [99]. A study of triple-negative breast cancer found that cancer cells overexpressing IRISOE secreted high levels of GM-CSF and activated STAT5, NF-kB, and ERK signaling in TAMs to enhance cancer cell proliferation, recruitment, and survival; M2 polarization of TAMs; and TGF-β1 expression and secretion. The inhibition of GM-CSF signaling attenuates TAM recruitment and M2 polarization, and also reduces the immunosuppressive capacity of IRISOE cells, thereby significantly reducing IRISOE tumor aggressiveness and regression through activated adaptive immune responses [99]. TAMs provide an immunosuppressive microenvironment for tumor cells, enabling them to fight against autoimmune death and fostering their survival and proliferation. To improve immunotherapy of tumors, understanding how TAMs induce immune microenvironment formation and targeting relevant targets may be beneficial.

**Fig. 3:**
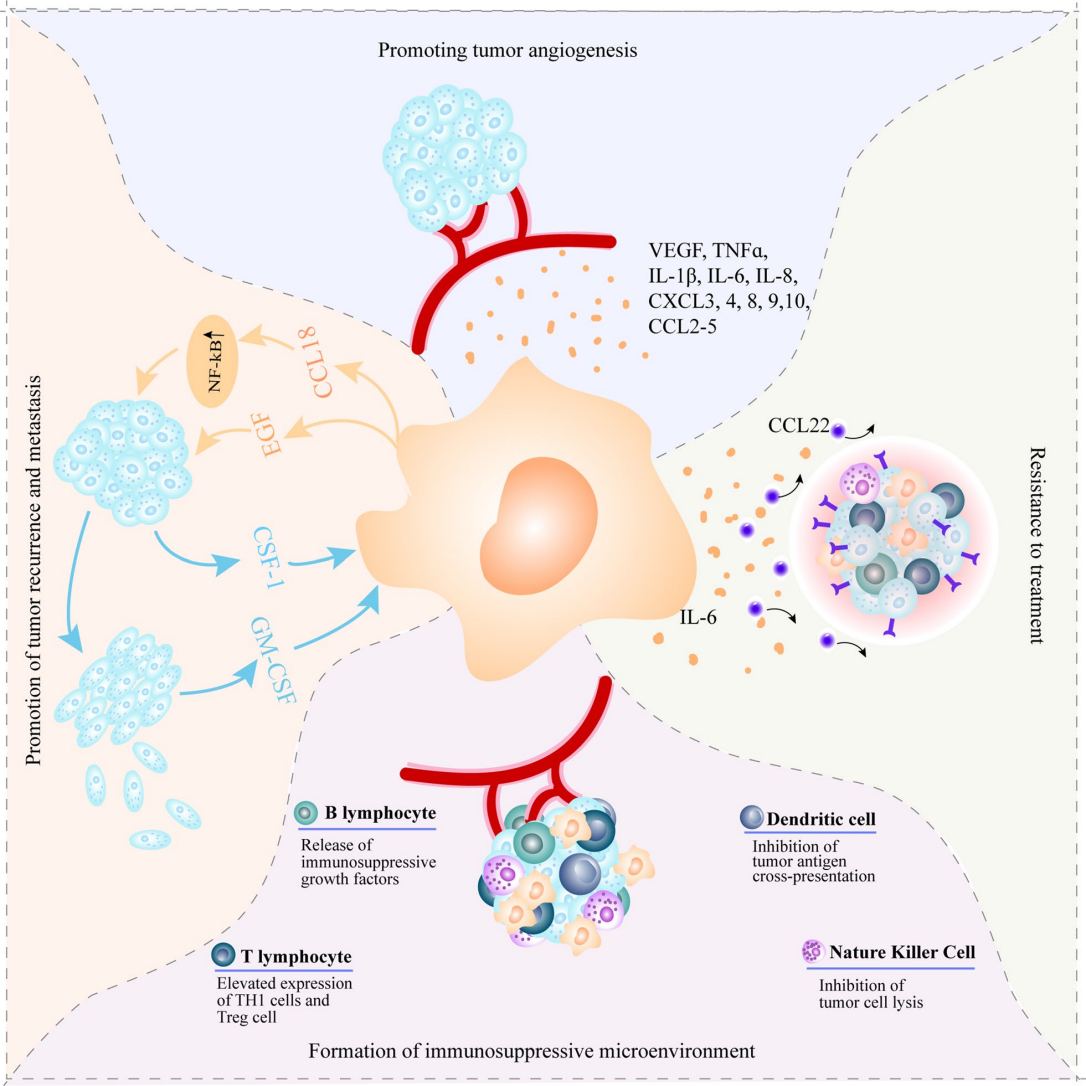
Mechanisms of TAMs promoting solid tumor progression. TAMs have the ability to promote tumor angiogenesis, assist tumor cell drug resistance, form a tumor immunosuppressive microenvironment and promote tumor recurrence and metastasis, creating a suitable environment for tumor cell growth and proliferation

In conclusion, as the most abundant immune cells in the TME, TAMs are key regulators in the TME and can promote tumor progression through various mechanisms. These mechanisms include the promotion of angiogenesis, promotion of tumor drug resistance, promotion of relapse and metastasis, and assistance in the formation of an immunosuppressive environment (Fig. 3).

## Strategies for targeting macrophages in the treatment of solid tumors

The most widely studied targeted therapies are the blocking of overexpressed genes, increased activation of tumor suppressor genes, targeting of tumor cell antigens, inducement of anti-tumor cell antigen activity, and inhibition or alteration of signals that control tumor cell growth and proliferation [100]. However, in recent years, an increasing number of studies have focused on the immunosuppressive effects of the TME, which affect the efficacy of tumor therapy, rather than relying solely on killing tumor cells [101].

TAMs are a major component of the TME and a mainstay in assisting tumor cells to form an immunosuppressive microenvironment. The modulation of TAMs is a new strategy for immunotherapy of solid tumors, and studies targeting TAMs have demonstrated a promising potential for future therapeutic strategies targeting TAMs in solid tumors. The aim of targeting TAMs in solid tumors is twofold: to reduce the number of TAMs in the TME as well as to alter the function of TAMs [100].

### Deletion of tumor-associated macrophages

TAMs prefer M2-type macrophages with anti-inflammatory and protumor effects. In the TME, numerous signaling molecules are involved in the process of macrophage polarization toward M2 phenotype. In malignant tumors, STAT3 is a critical factor driving the polarization of macrophages toward the M2 phenotype. ERK/STAT3 is the main signaling molecule in the lactate signaling pathway. In a breast cancer study, the inhibition of ERK/STAT3 signaling reduced lactate-induced M2 macrophage polarization and impeded breast cancer progression in vitro and in vivo [102]. In inflammatory breast cancer (IBC), strong expression of reactive STAT3 and IL-8 GRO chemokines causes high levels of monocyte recruitment and macrophage polarization factor expression. This promotes macrophage recruitment and polarization toward the M2 phenotype, which leads to the high infiltration of TAMs. This high infiltration of macrophages also feeds back to promote the expression of IL-8 and GRO chemokines, further contributing to the EMT of IBC [103].

Therefore, the inhibition of STAT3 signaling is crucial for improving the TME and inhibiting tumor progression. Many immunotherapeutic agents based on STAT3 targeting are in clinical trials for the treatment of solid tumors. Examples include IMX-110 for treating patients with advanced solid tumors (NCT03382340), disulfiram in combination with chemotherapy for treating refractory solid tumors or metastatic PDAC (NCT02671890), and a clinical trial evaluating the efficacy and safety of CKD516 in combination with durvalumab for treating refractory solid tumors (NCT04696848). In addition, several clinical trials of antibodies targeting STAT3 in solid tumor immunotherapy have been completed, such as OPB-111077 (NCT02250170 and NCT01711034) and OPB-51602 (NCT01423903 and NCT01184807).

STAT6 is another critical factor driving macrophage polarization toward M2 phenotype. Expression of the M2 gene has been demonstrated to be enhanced in STAT6-overexpressing macrophages. Activation of STAT6 mediates the transcriptional activation of M2 macrophage-specific genes, such as arginase 1 (Arg1), mannose receptor 1 (Mrc1), resistin-like α (Retnla, Fizz1), chitinase-like protein 3 (Chil3, Ym1), and the chemokine genes CCL17 and CCL24 [104]. Gene deletion or pharmacological inhibition of STAT6 significantly inhibits tumor growth and promotes anti-tumor immune responses in macrophages [105, 106].

Codiak et al. recently announced the initiation of a Phase I clinical trial of exoASO-STAT6 (NCT05375604), a novel engineered exosome candidate surface-loaded with antisense oligonucleotides (ASOs) targeting the STAT6 transcription factor. Specifically, exoASO-STAT6 selectively targets TAMs and precisely interferes with STAT6 signaling and inhibits M2 polarization, which induces anti-tumor immune responses [107]. Results from multiple *in-vitro* and *ex-vivo* experiments demonstrate that exoASO-STAT6 has potent single agent activity, including > 90% tumor growth inhibition and 50–80% complete remission (CR) rates. In the hepatocellular carcinoma (HCC) model, exoASO-STAT6 significantly downregulated STAT6 mRNA expression and effectively inhibited tumor growth; furthermore, 50% of mice had CR of tumor lesions. In addition, the combination of exoASO-STAT6 and PD-1 antibody significantly enhanced anti-tumor activity to the point that 75% of the tumor-bearing mice achieved CR [108]. This Phase I clinical trial will evaluate the safety, tolerability, biomarkers, and preliminary antitumor activity of exoASO-STAT6 in patients with advanced HCC, primary gastric cancer, and CRC. Initial data for the first phase are expected to be released in the first half of 2023.

In addition, both ANXA1 expression and S100A9 overexpression by naïve macrophages promote immunosuppression, resist inflammatory processes, and enhance the ability of macrophages to polarize toward the M2 phenotype [24, 109]. Therefore, targeting S100A9 and ANXA1 is a potential strategy for clearing TAMs [110, 111]. Besides inhibiting signaling molecules that induce M2 macrophage polarization and downregulating M2 macrophage production, studies have also elucidated the mechanisms of directly targeting the recognition signals of M2 macrophages and promoting the apoptotic process of M2 macrophages.

A study on lung cancer treatment using folic acid-modified cationic liposomes transporting the proapoptotic protein BIM, a mediator of Bcl-2-induced cell death, targeted tumor cells with high expression of folate receptor (FR) α and FRβ and M2-type macrophages in the mesenchyme [112]. F-PLP/pBIM was found to significantly inhibit lung cancer growth, reduce the number of tumor nodules, decrease the tumor weight, significantly reduce microvascular density, inhibit cell proliferation, promote apoptosis of tumor cells and M2 macrophages, significantly reduce the number of M2 macrophages, and alter the TME. F-PLP/pBIM had no significant toxic effects on mice [112].

Studies have also demonstrated that Melittin (MEL)-dKLA selectively binds to M2 macrophages, disrupts the cellular mitochondrial inner membrane, and induces apoptosis in M2 macrophages [23]. MEL-dKLA is a hybrid peptide composed of MEL and the proapoptotic peptide d(KLAKLAK)2 (dKLA). MEL preferentially binds to M2-type TAMs [113], while dKLA induces mitochondrial death after penetrating the cell membrane [114], thus causing apoptosis in M2 macrophages. This study also demonstrated the ability of MEL-dKLA to selectively bind CD206^+^ to M2 macrophages, which resulted in specific targeting of M2 macrophages while protecting M1 and DCs from anti-tumor functions.

In 2022, Sanchez-Paulete et al. reported that a novel CAR-T targeting F4/80 (F4.CAR-T) has recently been shown to effectively eliminate TAMs and release their immunosuppressive effects [115]. They found that F4.CAR-T not only effectively cleared TAMs and relieved TAM-induced immunosuppression, but also enhanced tumor antigen-specific T cell immune responses, thereby inhibiting the growth of a variety of tumors. Using a mouse model of NSCLC, F4.CAR-T significantly delayed progression and prolonged survival time in tumor-bearing mice. The anti-tumor effects of F4.CAR-T were also present in macrophage-rich ID8 ovarian cancer and PDAC tumor models, which significantly inhibited the growth of these two tumors [115].

### Inhibition of TAMs recruitment

The process of macrophage recruitment and differentiation is associated with local hypoxia, hyperlactate, and inflammatory states. In the TME, TAMs are mainly derived from BM monocytes and are recruited to the TME by chemokines such as CCL2, CCL5, CXCL12, and CSF-1 (or M-CSF). Once in the TME, TAMs can undergo phenotypic and functional changes in response to microenvironmental factors (e.g., hypoxia), making them more inclined to the M2 phenotype [116].

#### CCL2/CCR2 signals

CCL2 has been demonstrated to be a potent chemotactic agent for immune cells, such as monocytes, NK cells, memory T cells, and immature DCs, and mediates a variety of proinflammatory effects and neoangiogenesis [117]. The mobilization and uptake of circulating monocytes from BM to sites of inflammation are a process that is highly dependent on chemokine CCL2/CCR2 signaling [118]. Stromal cells, leukocytes, endothelial cells, and tumor cells in the TME have the ability to produce CCL2, driving the migration of circulating monocytes toward CC chemokines (e.g., CCL2). Driven by CCL2, TAMs accumulate toward the primary or secondary tumor sites [119]. Thus, CCL2/CCR2 inhibition keeps monocytes in the BM, leading to the depletion of the circulating cell pool and a reduction in the number of TAMs at primary and metastatic sites [120].

##### CCL2 neutralizing antibody—CNTO888

Carlumab, also known as CNTO888, is an IgG1k monoclonal antibody that binds with high affinity to CCL2. Specific binding of CNTO888 to CCL2 creates direct competition for the CCR2 binding site and inhibits the binding of CCL2 to the CCR2 [121]. The safety, efficacy, and pharmacokinetic parameters of CNTO888 alone or in combination with other commonly used chemotherapeutic agents in advanced solid tumors were evaluated in clinical trials (NCT00537368). The results indicated that CNTO888 was well tolerated in 44 patients with advanced solid tumors refractory to conventional treatment in a dosing treatment trial. Patients were treated with CNTO888 as a step-up dose from a starting dose of 0.3 mg/kg to a maximum planned dose of 15 mg/kg by intravenous injection, and the anti-tumor response was monitored according to PSA and carcinoembryonic antigen 125 (CA125) levels. The incidence of adverse events after CNTO888 administration was less than 37%, and the severity of adverse events was low and disappeared after discontinuation of treatment. However, free CCL2 levels were only temporarily suppressed after CNTO888 administration, with subsequent increases in free CCL2 concentrations even exceeding the levels of pretreatment baseline values, and total CCL2 (including the CNTO888-CCL2 complex) could increase more than 1,000-fold after treatment. Four patients maintained stable disease (SD) at 10.5 months (ovarian cancer), 5 months (prostate cancer), 7.2 months (ocular melanoma), and 15.7 months (neuroendocrine tumors), but none had an objective anti-tumor response [122]. Consequently, patients with solid tumors in the trial did not benefit overall from treatment with CNTO888.

Another phase I clinical trial of CNTO888 (NCT01204996) evaluated the safety, efficacy and pharmacokinetics of CNTO888 given in combination with standard chemotherapy regimens in 53 patients with solid tumors [123]. The 53 patients were divided into four treatment groups receiving CNTO888, docetaxel, gemcitabine, paclitaxel carboplatin, or polyethylene glycolated adriamycin hydrochloride liposomes. However, the combination of CNTO888 with the four chemotherapeutic agents neither enhanced the efficacy of these commonly used chemotherapeutic agents nor prolonged the duration of effective CCL2 blockade by CNTO888 [123].

In a phase II clinical trial study of CNTO888 for prostate cancer (NTC00992186), 46 patients with metastatic drug-resistant prostate cancer who had received hormone therapy or surgery and doxorubicin in combination with chemotherapy received CNTO888 as a single agent [124]. In this study, patients received intravenous CNTO888 (15 mg/kg) every 2 weeks. The results indicated that CNTO888 was generally well tolerated, with a small number of patients having mild to moderate adverse effects. After treatment with CNTO888 administration, free CCL2 levels decreased rapidly. The free CCL2 concentration, however, rebounded rapidly shortly after administration and quickly exceeded the pretreatment serum concentration, while the CCL2 level continued to rise after subsequent CNTO888 treatment. CNTO888 treatment did not produce any CR or partial remission (PR), and only 34% of patients had SD for more than 3 months [124].

In conclusion, the inability of CNTO888 to inhibit free CCL2 in patients for a sufficient duration may be a critical reason for the lack of clinically meaningful results of CNTO888 administration in this clinical trial. Further clinical studies are also needed to confirm the efficacies of CNTO888 in more types of solid tumors.

##### CCR2 inhibitor—MLN1202

MLN1202 is a humanized IgG1 antibody with specificity for the CCR2. A phase II clinical trial (NCT01015560) was performed to determine the efficacy of MLN1202 in 44 patients with bone metastases due to solid tumors [125]. MLN1202 (8 mg/kg) was administered intravenously as monotherapy on days 1, 15, and 29. The urinary type I collagen amino terminal peptide (U-NTX, a biomarker measuring the metabolic rate of osteocyte renewal), along with the anti-tumor activity and immune response, were evaluated in the patients’ urine. Consistent with CNTO888, MLN1202 treatment was well tolerated. After 43 days of MLN1202 treatment, only 14 of the patients (~ 32%) had significantly lower uNTX values, but its anti-tumor activity and effect on immune response were not revealed [125].

##### CCR2 inhibitor—CCX872

The CCR2 inhibitor CCX872-B was well tolerated by patients with PDAC when administered as a single oral dose (150 mg). In a phase Ib clinical trial (NCT02345408), all 50 patients enrolled had advanced unresectable PDAC (76% had metastatic PDAC). The study combined the CCR2 inhibitor CCX872 with the chemotherapy regimen FOLFIRINOX (5-fluorouracil, folinic acid, irinotecan, and oxaliplatin), and all patients received FOLFIRINOX once every 2 weeks (up to 12 doses) for 24 weeks in combination with 150 mg of CCX872 once or twice daily. At month 18, the overall patient survival rate was observed to be 29% [126]. The results of the study revealed a disease control rate (DCR) of 78% and an objective response rate (ORR) of 30–37% for CX872-B plus FOLFIRINOX, with no safety issues with CCX872-B. This suggested that CCX872 had positive efficacy in these patients [127].

#### CSF-1/CSFR signals

The colony-stimulating factor 1 receptor (CSF-1R) signaling pathway drives the recruitment of TAMs to the TME and promotes their differentiation to a protumor phenotype [128]. CSF-1 also promotes immunosuppression through stimulating the differentiation of MDSCs and the selective activation of TAMs expressing CSF-1R and MHC-II. Immune cell infiltration with high expression of CSF-1R in the TME is usually associated with immune dysfunction and enhanced immune resistance, such as poor prognosis in ovarian cancer, breast cancer, endometrial cancer, PDAC, and lymphoma [129–132]. Blocking the CSF1/CSF-1R axis is the most established method for reducing the infiltration of TAMs. This approach reduces their number by blocking monocyte differentiation as well as decreases the survival rate of existing TAMs [100, 133].

The tyrosine kinase inhibitor PLX3397 was found to possess the ability to inhibit CSF-1R in a mouse model for the treatment of melanoma. PLX3397 is currently used clinically in the treatment of patients with glioblastoma, breast cancer, and other cancers to inhibit CSF-1R, leading to reduced M2 macrophage recruitment [134]. A phase 1 Study of the CSF-1R inhibitor LY3022855 in metastatic breast cancer or metastatic debulking-resistant prostate cancer demonstrated high tolerability [135].

Regimens demonstrated that combined with anti-CSF-1R and CD40 agonists significantly reduce TAMs and Foxp3^+^ Tregs [136]. And this combination increases the maturation and differentiation of proinflammatory macrophages and DCs, drives the efficient initiation of effector T cells in the draining lymph nodes, and results in enhanced tumor-infiltrating effector T cell attack against tumor antigens. Furthermore, studies have suggested that the combination therapy may simultaneously eliminate suppressive immune populations and maintain endogenous anti-tumor immune responses, successfully halting cancer progression [133, 136]. Inhibition of the PI3k-γ and CSF-1R dual signaling pathway repolarized M2-type TAMs to the M1-type and reduced the infiltration of MDSCs. This dual inhibition led to enhanced reprogramming of TAMs and tumor suppression through various mechanisms [137]. PI3K, which is mainly expressed in myeloid cells and is a key signaling molecule in many critical signaling pathways, controls the critical switch between stimulation and suppression of immune function and plays an important role in TAM-mediated immunosuppression [138]. Drugs targeting PI3K have started to enter clinical trials, but thus far most mainly selectively target PI3K isoforms. This selective action alleviates the toxic side effects of the drug to a certain extent and improves the tolerance of patients.

#### CXCL12/CXCR4 signals

Another pathway involved in macrophage recruitment is the CXCL12/CXCR4 axis. Both tumor cells and TAMs can secrete CXCL12 (stromal cell-derived factor-1; SDF), which is a unique receptor for CXCR4 [13]. The CXCL12/CXCR4 signaling axis is involved in the recruitment of monocytes in tumors. Chemokine CXCL12 recruits CXCR4-expressing monocytes into the TME and induces their differentiation toward immunosuppressive TAMs, thereby promoting tumor growth, invasion, and metastasis [139, 140]. As such, targeting the CXCL12/CXCR4 axis can block TAM recruitment and reduce the infiltration of TAMs.

Currently, 22 targeted CXCR4 drugs are in active development, which are mainly small molecule antagonists, but some are antibody drugs, gene therapy, and CAR-T therapies. Eighteen of these drugs are for the treatment of hematological malignancies or solid tumors. Mozobil (Plerixafo), a small molecule antagonist targeting CXCR4, was launched in China in 2018. It is primarily used in combination with granulocyte colony-stimulating factor (G-CSF) to mobilize hematopoietic stem cells for the treatment of non-Hodgkin's lymphoma and multiple myeloma. On January 18, 2022, Motixafortide (BL-8040), a synthetic peptide CXCR4 antagonist, underwent a pre-new drug application (pre-NDA) meeting and is expected to receive marketing approval in 2022 primarily for the treatment of breast cancer.

### Release of the phagocytosis by TAMs

**Fig. 4:**
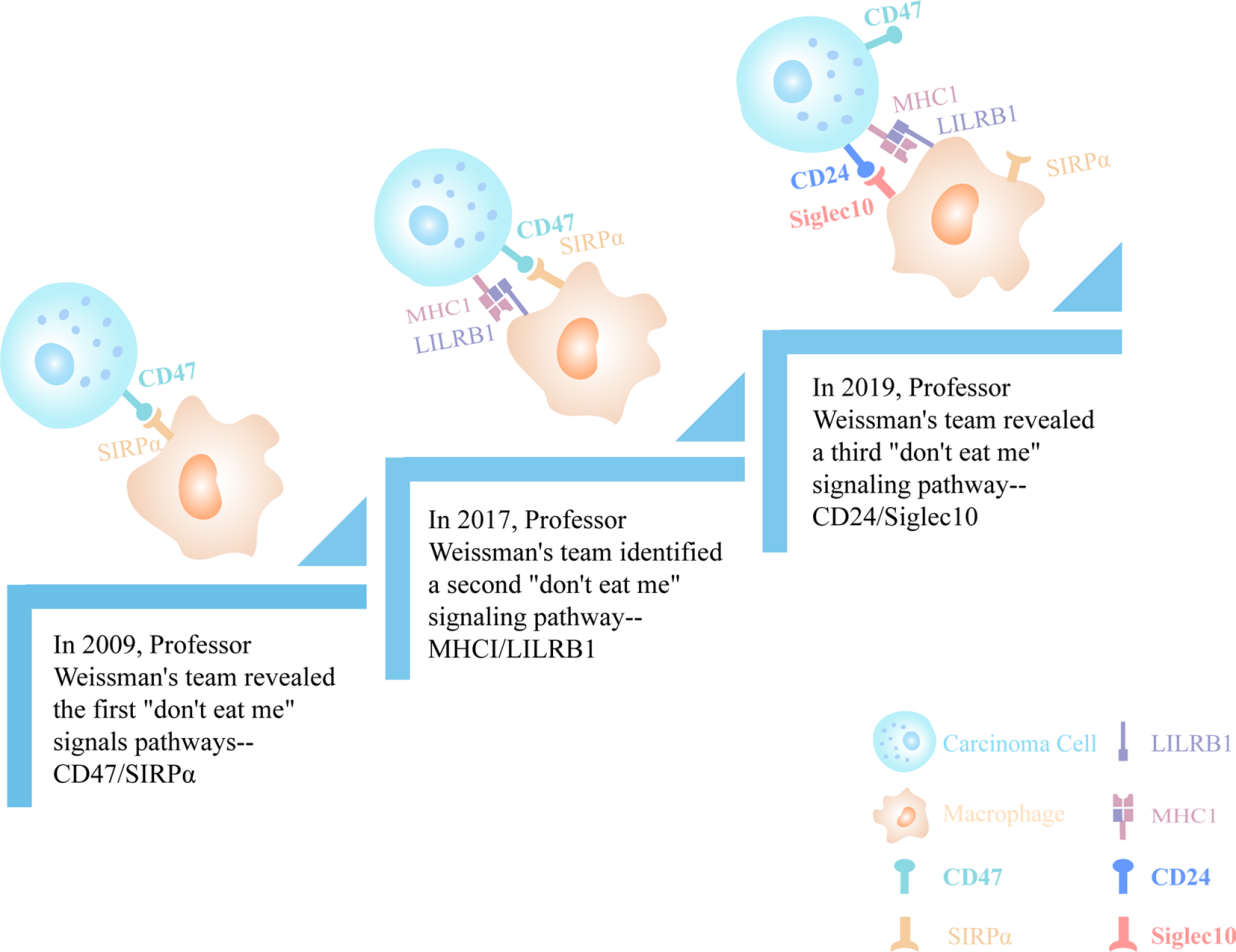
The development process of the “Don’t eat me” signal

In tumor tissue, macrophage phagocytosis is inhibited by “don’t eat me” signals on the surface of tumor cells (Fig. 4). Therefore, identifying “don’t eat me” signals and downregulating their expression represent an effective method for promoting the phagocytosis of TAMs.

#### “Don’t eat me” signal—CD47/SIRPα

CD47 is a membrane protein widely distributed on the surface of various cells, including tumor cells. The signaling regulatory protein (SIRPα) is a ligand for CD47 and is mainly expressed in macrophages [141]. The interaction of CD47 with SIRPα inhibits the phagocytosis of macrophages in cells expressing CD47. Thus, CD47 is considered a “don’t eat me” signal [142]. Under normal conditions, the expression of CD47 and SIRPα is in equilibrium. By contrast, elevated CD47 expression on the surface of tumor cells assists the cells in evading phagocytosis by macrophages [44]. Therefore, inhibiting the expression of CD47 molecules in tumor cells or blocking the recognition pathway between CD47 and SIRPα can reactivate the phagocytosis of tumor cells by macrophages and inhibit the progression of tumor progression [141].

In recent years, CD47/SIRPα has become another attractive target for tumor immunotherapy after PD-1/PD-L1 targets. Targeting CD47 to enhance macrophage phagocytosis has emerged as a promising strategy for tumor immunotherapy. Currently, anti-tumor therapeutics targeting the CD47/SIRPα axis are emerging, including monoclonal antibodies (mAbs), fusion proteins, bispecific antibodies (BsAbs), Antibody–Drug Conjugates (ADCs), small molecule drugs, and CAR-T. Among them, CD47 mAbs are the most common, followed by BsAbs and fusion proteins [143–145].

##### Monoclonal antibodies targeting CD47/SIRPα

The mAbs of CD47 are divided into three main categories based on their form of action. The first class binds to CD47 on the surface of tumor cells, erythrocytes, and platelets, represented by Forty Seven’s Hu5F9-G4 (5F9, Magrolimab). Hu5F9-G is a humanized IgG4 antibody that targets CD47 with high affinity, but its binding to erythrocytes and platelets can cause anemic reactions [146]. Currently, 5F9 is in clinical trials for patients with solid tumors (NCT02216409). Based on 100% erythrocyte CD47 saturation tolerance and receptor occupancy studies to avoid the hematotoxicity of 5F9, in a first phase I clinical trial of Hu5F9-G4 in patients with advanced cancer, 1 mg/kg was selected as the starting dose to trigger the clearance of aged erythrocytes in vivo and stimulate reticulocyte maturation and differentiation to produce fresh young erythrocytes. In the subsequent group, patients received maintenance doses ranging from 3 mg/kg to 45 mg/kg, with most toxicity being mild to moderate. The most common toxicity was an expected, targeted, mild, transient anemia that resolved when administered at the initial and maintenance doses in Cycle 1. In addition, a clinical trial is ongoing for 5F9 in combination with Cetuximab for solid tumors and advanced CRC (NCT02953782); another ongoing program is 5F9 in combination with Avelumab for the treatment of patients with solid tumors that have progressed within 6 months after prior platinum-based chemotherapy and patients with ovarian cancer that is primary resistant to immune checkpoint inhibitors (NCT03558139) [147, 148].

The second category is antibodies obtained by specific screening that do not bind or bind weakly to erythrocytes. Compared with Class I antibodies, these antibodies have a theoretically higher safety profile and do not require modified clinical dosing regimens to reduce the associated hematological toxicity. This group is represented by antibodies such as AK117 (Ligufalimab) and TJC4 (Lemzoparlimab). AK117 is a novel IgG4 mAb targeting CD47 that uses the Ig4 isoform and is engineered to minimize Fc effects [149]. Compared with other anti-CD47 mAbs, AK117 binds more weakly to human erythrocytes than to tumor cells [150]. This gives it a potential prophagocytic activity and a good hematological safety profile. AK117 has demonstrated potential anti-tumor activity in both *in-vitro* and *in-vivo* nonclinical studies. The *in-vitro* tests did not cause significant agglutination of human erythrocytes, while *in-vivo* tests in a macaque exhibited minimal effects on anemia, with only minor erythrocyte changes [150]. In a phase I study evaluating the safety of AK117 in advanced or metastatic solid tumors (NCT04349969), 15 patients were enrolled in phase 1a as of February 15, 2021, for DLT evaluation of the ongoing 30 mg /kg cohort. The results revealed that AK117 was safe and well tolerated up to 20 mg/kg QW; no IRRs or severe TRAEs were observed; and no hematological TRAEs occurred except in patients with baseline G1 anemia taking 10 mg/kg AK117 [151]. Another phase I study demonstrated similar results in 27 solid tumors receiving CD47 mAbs, AO-176 [152]. This study also reported that 7 patients had SD as a best response, with 2 patients (endometrial carcinoma, gastric cancer) on study for more than 6 months.

The third category is CD47mAbs that do not have binding capacity in blood and circulation, such as the ADG153 SAFEbody. When these antibodies enter the TME, they can be cleaved to expose binding epitopes and activate their binding activity under the cleavage of tumor-specific enzymes [153]. However, preclinical and clinical trials of these antibodies have not been conducted in solid tumors.

In conclusion, the safety of mAbs, targeting CD47 in the treatment of solid tumors has been demonstrated. However, the efficacy of CD47 mAbs alone is not very prominent in solid tumors. Further studies are required to confirm this and the selected clinical trials of mAbs, targeting CD47/SIRPα are shown in Table 2.

**Table 2:** Clinical trials of CD47/SIRPα targeted agents in solid tumors

mAbs	Inhibitor type	Conditions	Monotherapy or Combination	Number of patients recruited	Phases stage	Clinical trial NO
Hu5F9-G4 (Magrolimab)	Humanized, IgG4	Solid Tumor	Monotherapy	88 participants	Phase I	NCT02216409
AK117(Ligufalimab)	Humanized, IgG4	Neoplasms Malignan	Monotherapy	162 participants	Phase I	NCT04728334
		Neoplasms Malignan	Monotherapy	159 participants	Phase I	NCT04349969
		Metastatic and locally advanced TNBC	Combination	80 participants	Phase II	NCT05227664
		Advanced malignant tumors	Combination	130 participants	Phase I/II	NCT05235542
		Advanced malignant tumors	Combination	114 participants	Phase I/II	NCT05229497
		Advanced malignant tumors	Combination	160 participants	Phase I/II	NCT05214482
		Metastatic colorectal cancer	Combination	114 participants	Phase II	NCT05382442
AO-176	Human, IgG4	Solid tumor	Combination	183 participants	Phase I/II	NCT03834948
SGN-CD47M	Humanized, IgG4	Solid tumor	Monotherapy	16 participants	Phase I	NCT03957096
CC-9002	Humanized, IgG4	Hematologic neoplasms	Monotherapy, or combination	60 participants	Phase I	NCT02367196

##### Bispecific antibodies targeting CD47/SIRPɑ

BsAbs are synthetic antibodies with two specific antigen binding sites. The China Drug Clinical Trial and Information Disclosure Platform (http://www.chinadrugtrials.org.cn/) presents more than 80 BsAbs, while a clinical trial website platform (https://clinical.trials.gov/) showcases more than 200. Among them, BsAbs targeting the CD47/SIRPα axis are the main research focus [154]. Anti-CD47/SIRPα BsAbs are classified according to the antigen that they target; thus, they can be classified as BsAbs targeting tumor antigens (PD-L1, CD20, CD19, mesothelin [MSLN], Claudin18.2, and Her2), BsAbs targeting immune cells (PD-1, CD40, and 41BB), BsAbs targeting cytokines or receptors (CSF-2 receptor/VEGF), and BsAbs targeting cytokines or receptors (CSF-2 receptor/VEGF) among others. Most BsAbs targeting the CD47/SIRPα axis reduce anemia by modulating the affinity of the two binding structural domains, as demonstrated by the relative reduction in affinity for targeting CD47.

Partly based on targeting the CD47/SIRPα axis, BsAbs are also currently in preclinical or clinical trials for the treatment of solid tumors (Tables 3, 4, 5). These include CD47/PD-1 (PD-L) BsAb, CD47/HER2 BsAb, CD47/GPC3 BsAb, CD47/EGFR BsAb, SIRPα/VEGFR1 BsAb, CD47/MSLN BsAb, SIRPα/CD40L BsAb, SIRPα/CTLA4 BsAb, CD47/DLL3 BsAb, and Claudin18.2/CD47 BsAb [155–165].

**Table 3:** Preclinical studies of CD47 BsAb in solid tumors

Targets	Type of diseases	References
CD47/GPC3	HCC	[156]
CD47/EGFR	EGFR + tumors	[157]
SIRPα/VEGFR1	NSCLC, Glioblastoma	[158, 172]
CD47/MSLN	MSLN + tumors	[159]
SIRPα/CD40L	Solid tumors (mouse CT26 tumor model)	[160]
SIRPα/CTLA4	Solid tumors	[161]
CD47/DLL3	SCLC Neuroendocrine cancers	[162]
Claudin18.2/CD47	gastric cancer Gastroesophageal junction (GEJ) cancer Pancreatic cancer	[163]

**Table4:** Clinical trials of CD47 BsAb in solid tumors

BsAb	IgG subclass	Target	Conditions	Monotherapy or Combination	Number of patients recruited	Phases stage	Clinical trial NO
HX009	IgG4	CD47 + PD-1	Advanced solid tumor	Monotherapy	21 participants	Phase I	NCT04097769
			Advanced solid tumor	Monotherapy	210 participants	Phase II	NCT04886271
IBI322		CD47 + PD-L1	Advanced solid tumor	Monotherapy	36 participants	Phase I	NCT04912466
			Advanced malignant tumors lymphomas	Monotherapy	51 participants	Phase I	NCT04338659
			Advanced malignancies	Monotherapy	218 participants	Phase I	NCT04328831
			Small cell lung cancer	Combination	40 participants	Phase II	NCT05296603
			Non small cell lung cancer	Combination	80 participants	Phase II	NCT05296278
			Myeloid tumor	Combination	124 participants	Phase I	NCT05148442
6MW3211		CD47 + PD-L1	Advanced malignant neoplasm	Monotherapy	272 participants	Phase I/II	NCT05048160
PF-07257876	IgG1	CD47 + PD-L1	NSCLC SCCHN and ovarian cancer	Monotherapy	90 participants	Phase I	NCT04881045
IBC0966		CD47 + PD-L1	Advanced malignant tumors	Monotherapy	228 participants	Phase I/II	NCT04980690
IMM2902	IgG1	CD47 + HER2	HER2 + advanced solid tumors	Monotherapy	40 participants	Phase I	NCT05076591

**Table 5:** Clinical trials of SIRPα targeted agents in solid tumors

Code	Inhibitor type	Conditions	Monotherapy or Combination	Number of patients recruited	Phases stage	Clinical trial NO
CC-95251	Human	Advanced solid and hematologic cancers	Monotherapy, or Combination	230 participants	Phase I	NCT03783403
BI 765,063 (OSE-172)	Humanized IgG4	Solid tumors	Combination	18 participants	Phase I	NCT04653142
		Solid tumor, adult	Combination	116 participants	Phase I	NCT03990233
		HNSCC melanoma NSCLC	Combination	22 participants	Phase I	NCT05068102
		HNSCC	Combination	150 participants	Phase I	NCT05249426
Evorpacept (ALX148)	mutated SIRPα-Fc IgG1	HNSCC	Combination	168 participants	Phase II	NCT04675333
		HNSCC	Combination	183 participants	Phase II	NCT04675294
		HER2 + gastric cancer	Combination	450 participants	Phase II/III	NCT05002127
		Metastatic cancer; solid tumor; advanced cancer; NHL	Combination	174 participants	Phase I	NCT03013218
		Microsatellite stable metastatic colorectal cancer	Combination	80 participants	Phase II	NCT05167409
		HER2-expressing cancers	Combination	93 participants	Phase I/II	NCT05027139
TTI-621	SIRPα-Fc IgG1	R/R solid tumors and mycosis fungoides	Monotherapy,or combination	174 participants	Phase I	NCT02890368
		Hematologic malignancies solid tumor	Monotherapy, or combination	250 participants	Phase I	NCT02663518
		Leiomyosarcoma	Combination	80 participants	Phase I/II	NCT04996004
TTI-622	SIRPα-Fc IgG4	Platinum-resistant ovarian cancer	Combination	50 participants	Phase I/II	NCT05261490

CD47/PD-1 BsAb: The interaction of anti-PD-1/PD-L1 with anti-CD47 in immunosuppression mediated by TAMs implies targeting both innate and adaptive dual immune checkpoints, suggesting that combined therapies improve survival more than monotherapy [166]. Thus, CD47/PD-1(PD-L1) BsAb may maximize the effect of anti-tumor therapy and trigger longer-lasting therapeutic responses [167].

The recombinant humanized antibody fusion protein HX009 is a BsAb that binds to both CD47 and PD-1, and Phase I (NCT04097769) and Phase II (NCT04886271) clinical trials of the drug are underway. The results of the phase I trial indicated that treatment-related adverse reactions occurred in 10 of the 21 patients treated (47.6%), and most AEs were grade 1 or 2. Furthermore, three patients had PR, and six patients had SD, which demonstrated satisfactory treatment results [155].

CD47/PD-L1 BsAb: IBI322, a BsAb targeting CD47 and PD-L1, selectively binds to CD47 and PD-L1 co-expressing tumor cells on the one hand, thereby attenuating CD47 activity in monovalent binding and blocking PD-L1 activity in bivalent binding, stimulating strong anti-tumor activity. On the other hand, IBI322 effectively accumulates in PD-L1 positive tumors, blocks PD-1 and PD-L1 binding, activates CD8^+^ T cells, stimulates adaptive immune responses, and exhibits synergistic activity in inducing complete tumor regression in vivo [156, 168]. PD-L1 is expressed in tumor cells and IBI322 has a stronger affinity for PD-L1 than CD47, suggesting that IBI322 can bind more selectively to tumor cells than to normal cells. Preliminary studies suggest that IBI322 binds to PD-L1-positive tumor cells more than to red blood cells; therefore, it does not induce hemagglutination and has a stronger safety profile [169]. In addition, IAB, another “Knobs-into-holes”–based dual-targeting fusion protein that targets CD47 and PD-L1, also demonstrated to have high safety and anti-tumor activity in tumor-bearing mice [170].

CD47/HER2 BsAb: Previous studies have demonstrated that trastuzumab in combination with CD47 mAb completely eliminates tumors in a mouse model of human HER2^+^ breast cancer transplantation tumors [171, 172]. Based on this finding, the investigators (ImmuneOnco Shanghai Biomedical Co., Ltd.) prepared CD47/HER2 BsAb-IMM2902 using the “mAb Trap” technique. IMM2902 allows the drug to bind preferentially to tumor cells through the high-affinity activity of HER2, and it simultaneously exhibits the characteristics of not binding to human erythrocytes and avoiding “antigenic sink,” thus greatly enhancing the specific synergistic effect of the dual target on tumors. In nonhuman primates, different doses of IMM2902 had no effect on hemagglutination and no significant hematotoxicity [157]. The established BT-474 breast cancer and NCI-N87 gastric xenograft tumor models exhibited complete tumor elimination in *in-vivo* efficacy studies, even at doses as low as 3.5 mg/kg. Significantly, in a Herceptin-resistant breast tumor model, HCC-1954 tumors, IMM2902 also yielded strong anti-tumor activity at a dose of 10 mg/kg [157]. IMM2902 has been approved to enter clinical trials for the primary indication of HER2-positive breast, gastric, lung, and other advanced solid tumors to assess its efficacy and safety in HER2^+^ advanced solid tumors (NCT05076591).

CD47/GPC3 BsAb: GPC3 is an HCC-associated antigen specifically expressed in HCC, while the expression of CD47 in HCC inhibits its phagocytosis. As a result, a new BsAb, namely GPC3/CD47 biAB, was generated. In-vitro and in-vivo experiments revealed the safety profile of GPC3/CD47 biAb, its long half-life, and its more pronounced affinity for dual-antigen-expressing tumor cells, which highlight the advantages of its anti-tumor activity [158]. In an hCD47/hSIRPɑ humanized mouse model, the serum half-life of GPC3/CD47 biAb was prolonged without hematological toxicity; in in-vitro experiments, GPC3/CD47 biAb enhanced the Fc-mediated effector function against dual antigen-expressing HCC cells. Moreover, in a xenogeneic HCC model, GPC3/CD47 biAb was superior to monotherapy as well as in combination with anti-CD47 and anti-GPC3 mAbs. These results suggest that GPC3/CD47 biAb will further improve cancer treatment with GPC3/CD47 dual antigen expression [158].

CD47/EGFR BsAb: In addition to being expressed on tumor cells, CD47’s widespread expression on normal cells limits the clinical efficacy of CD47 mAbs [173]. EGFR is a cell surface target antigen that conducts oncogenic signals and is overexpressed in various malignancies. The generation of BsAb CD47xEGFR-IgG1 blocks CD47 expressed on the surface of cancer cells in an EGFR-directed manner, effectively reducing the targeting/nontumor effects [159]. Studies demonstrated that BsAb CD47xEGFR has enhanced overall affinity for CD47/EGFR double-positive cancer cells and selectively induces the phagocytosis and immune antigenic processing of double-positive cancer cells; it also enhances the elimination of tumor cells and promotes adaptive anti-cancer immune responses, thereby improving the selectivity and therapeutic efficacy of the CD47/SIRPα checkpoint inhibition approach in EGFR-overexpressing malignancies [159].

SIRPα/VEGFR1 BsAb: The association of VEGF/VEGFR inhibitor VEGFR1-Fc with CD47-blocking fusion protein produces synergistic anti-tumor efficacy. Targeting CD47 was demonstrated to trigger the macrophage-mediated clearance of recurrent NSCLC cells, and targeting both VEGF and CD47 via the VEGFR1-SIRPα fusion protein induces macrophage infiltration and enhances the ability to destroy anti-tumor cells and sensitize NSCLC to angiogenesis inhibitors and CD47 blockade [160]. VEGFR1D2-SIRPɑD1, consisting of the second extracellular structural domain of VEGFR1 (VEGFR1D2) and the first extracellular structural domain of SIRPɑ (SIRPɑD1), exerts its potential anti-tumor effects in glioblastoma treatment by inhibiting VEGF-induced angiogenesis and activating macrophage-mediated phagocytosis [174].

CD47/MSLN BsAb: MSLN is a cell surface glycoprotein that is overexpressed in a variety of solid malignancies, including gastric, lung, mesothelioma, pancreatic, and ovarian cancers [175]. BsAbs are formed by combining the high-affinity binding arm of MSLN with the blocking CD47 arm. They are designed to target MSLN and CD47 dual-positive tumor cells. In an in-vitro phagocytic killing assay, BsAb targeting MSLN/CD47 exhibited stronger ADCP activity by targeting the proximal epitope of the MSLN membrane than the distal region of the membrane, optimized ADCC activity by enhancing FcγR-IIIA activation, and enhanced ADCP through a more effective blockade of CD47/SIRPα. This BsAb also exhibited superior anti-tumor activity in a xenograft tumor model [161].

SIRPα/CD40L BsAb: Preclinical studies demonstrated that CD40 signaling enhances CD47/SIRPɑ blockade on the phagocytosis of tumor cells by macrophages and the cross-presentation of tumor antigen CD8^+^ T cells by DCs [176]. The novel BsAb SIRPɑ-Fc-CD40L is a two-sided fusion protein capable of binding the extracellular structural domains of SIRPɑ and CD40L and also of binding a central Fc structural domain [162]. SIRPɑ-Fc-CD40L, which binds CD47 and CD40 with high affinity, potently enhances anti-tumor immunity by synergizing type I IFN responses through CD40 stimulation with CD47/SIRPɑ blockade. In a cynomolgus macaque model, SIRPɑ-Fc-CD40L stimulated the elevation of multiple serum cytokines and the marginalization of CD40^+^ B cells in a dose-dependent manner; however, no signs of hemolysis, hemagglutination, or thrombocytopenia were observed in vitro or in nonhuman primates [162]. Furthermore, mouse-derived SIRPɑ-Fc-CD40L exhibited superior anti-tumor activity and long-term immune effects over CD47 mAbs and CD40 mAbs in a mouse CT26 tumor model. SIRPɑ-Fc-CD40L synergized with PD-1 and CTLA4 ICIs to increase DC activity, upregulate type I interferon-gamma response, and enhance macrophage-mediated phagocytosis in vitro, as demonstrated by the significantly higher phagocytic activity of SIRPɑ-Fc-CD40L in combination with rituximab in lymphoma cell lines and mouse tumor models (CD20^+^WEHI3 and A20) compared with in combination with CD47 mAb and rituximab [162].

SIRPα/CTLA4 BsAb: CTLA-4 is an immune checkpoint protein highly expressed on Tregs in the TME [177, 178]. An anti-CTLA-4 antibody targeting Tregs binds a heterodimer of SIRPα that selectively blocks CD47 on Tregs in tumors by blocking “don’t eat me” signaling and enhancing “eat me” signaling to deplete Tregs in an Fc-dependent depletion of Tregs. In MC38 and CT26 mouse colon cancer models, anti-CTLA-4 × SIRPα preferentially depleted immunosuppressive Tregs of ICOS^high^ in the TME and enhanced immunity against solid tumors. This BsAb has a lower affinity for individual targets and therefore exhibits lower toxicity than anti-human CTLA-4 [163]. In the MC38 mouse colon cancer model, a single low dose administered systemically for 5 days had a half-life of more than 21.4 h and was preferentially concentrated in tumor tissues compared with normal tissues and organs. In addition, it promotes IFN-γ-dependent T cell responses, which reduces the tumor burden in mice [163].

CD47/DLL3 BsAb: Recently, the FDA granted orphan drug designation to PT217 as a potential treatment option for patients with small cell lung cancer (SCLC). PT217 is a first-in-class BsAb with Fc effect, designed to target DLL3 and CD47 in patients with SCLC and other neuroendocrine cancers. DLL3 is highly expressed restrictively in SCLC cells and could be an attractive target for SCLC immunotherapy. PT217 is intended to mediate potential antibody-dependent cytotoxicity of NK cells against tumor cells and to block the interaction of CD47 with SIRPα [164]. PT217 exhibited potential inhibitory activity in preclinical mouse xenograft models, and toxicity studies in nonhuman primates and rats supported the first human clinical trials. At PT217 doses of 3, 10, and 30 mg/kg, the erythrocyte, hemoglobin, leukocyte, and reticulocyte counts remained within normal limits during the first 21 days of treatment in the nonhuman primate model. In a presentation at the 2022 AACR Annual Meeting, researchers presented the structure of a phase 1 dose-escalation trial for studying the role of PT217 in patients with DLL3 positive SCLC, large cell neuroendocrine carcinoma, and neuroendocrine prostate cancer. A dose-escalation-guided 3 + 3 design will evaluate PT217 at five different dose levels administered weekly [164].

Claudin18.2/CD47 BsAb: On June 15, 2022, Phanes Therapeutics announced that its Claudin18.2/CD47 BsAb PT886 had received FDA clinical trial approval to conduct a phase I clinical trial in patients with gastric cancer, gastroesophageal junction cancer, and PDAC. PT886 is an anti-CLDN18.2/anti-CD47 BsAb with a natural IgG structure [165]. It is achieved through two mechanisms of tumor killing: (1) the CD47/SIRPα axis is blocked and macrophages are stimulated to phagocytose tumor cells; and (2) the functional Fc of BsAbs mediates the potential effects of NK cell antibody-dependent cellular cytotoxicity (ADCC) and macrophage antibody-dependent cellular phagocytosis (ADCP) [165]. PT886 has a high affinity for CLDN and a low affinity for CD47, which gives it a high safety profile and the ability to bind specifically to CLDN-expressing tumor cells, while binding weakly or not to CD47-expressing normal cells. In *in-vitro* phagocytosis assays, CLDN18.2 binding resulted in a stronger stimulation of phagocytic activity by PT886. The anti-tumor activity of PT886 was also demonstrated in an *in-vivo* pancreatic cancer xenograft model, where PT886 treatment resulted in complete tumor clearance at doses as low as 1 mg/kg. PT886 also has a good safety profile in nonhuman primate and demonstrates manufacturability similar to conventional monoclonal antibodies [165].

In summary, most of the clinical trials of BsAbs, targeting CD47 are in phase I clinical trials, and their value in solid tumors has yet to be verified.

##### Combined therapy targeting CD47/SIRPɑ

A recent study by a team led by the Stanford University School of Medicine found that the combination of anti-GD2 and CD47 antibodies exhibited strong synergistic effects in neuroblastoma and other GD2^+^ malignancies, leading to the increased infiltration of TAMs within the tumor and the mediation of its durable anti-tumor response by GD2-specific factors [179]. GD2 belongs to the ganglioside sphingolipid class and is expressed in solid tumors, such as neuroblastomas and osteosarcomas, whereas it is restrictedly expressed in other normal tissues [180]. The GD2 antibody blocks the binding of GD2 to Siglec-7, eliminates the “don’t eat me” signal expressed by tumor cells to macrophages and enhances the phagocytic activity of TAMs. Furthermore, its combination with CD47 antibody can stimulate macrophages to produce more powerful anti-tumor phagocytic effects. A clinical trial using this combination to treat patients with neuroblastoma and osteosarcoma (NCT04751383) is underway [179].

##### Trispecific antibodies targeting CD47/SIRPɑ

Single-domain VHH trispecific antibodies have been developed. Trispecific antibodies have multiple mechanisms of action and can act alone or in combination to attack tumors [181]. KB-436, a trispecific antibody targeting dopamine receptor 2 (DRD2), PD-1, and CD47, is in preclinical trials. DRD2 is a G protein-coupled receptor that is upregulated in many types of cancer and is associated with decreased patient survival. The VHH module (anti-DRD2, anti-PD-1, and anti-CD47) of KB-436 mediates multiple mechanisms of action. Anti-DRD2 VHH induces intracellular signal transduction; anti-PD-1 VHH restores T-cell function; and anti-CD47 VHH recruits T cells without extensive activation and blocks the interaction of CD47 with SIRPα. The anti-tumor efficacy of KB-436 was tested in vivo in an immuno-oncology xenograft model formed by human SCLC and several other solid tumors reconstituted from human peripheral blood mononuclear cells (PBMC) or CD34^+^ hematopoietic stem cells. The treatment inhibited tumor growth, enhanced the *in-vivo* effects of cisplatin treatment on the less chemosensitive NCI-H69 variant, blocked tumor metastasis formation in CD34^+^ humanized NCG mice carrying established NCI-H69 tumors, and increased survival in a mouse model of tail vein metastasis. The half-life of trispecific KB-436 in mice is approximately 5 days, which is consistent with that of other antibodies. It is produced in high yields (6 g/L) in manufactured cell lines with a conventional purity of 99% and exhibits significantly high stability in accelerated stability tests [181]. In conclusion, the aforementioned data support the clinical development of KB-436 in the treatment of advanced metastatic solid cancers and offer new possibilities for the clinical treatment of metastatic solid tumors.

#### “Don’t eat me” signal – MHC1/LILRB1

In November 2017, Professor *Irving Weissman’s* team published a study in *Nature Immunology* that revealed a second “don’t eat me” signal on tumor cells, namely major histocompatibility complex (MHC) I [182]. The study indicated that the inhibitory receptor LILRB1 on the surface of macrophages can bind to the β2 microglobulin (β2M) component of MHC I, which is widely present on the surface of tumor cells. This binding is consistent with the CD47 pathway, which blocks the phagocytosis of tumor cells by macrophages. The study demonstrated that when LILRB1 protein was inhibited or not expressed, the phagocytosis of tumor cells by macrophages in tumor-bearing mice was enhanced and their survival time was prolonged by 70% [182]. The inhibition of this molecule along with the administration of anti-CD47 mAb significantly increased the phagocytosis and killing ability of macrophages against tumor cells; yet, the inhibition of LILRB1 did not damage normal tissue cells in vivo. This approach of targeting macrophages in combination with current immunotherapy that enhances the anti-cancer activity of T cells has potential anti-cancer activity [182].

#### “Don’t eat me” signal–CD24/Siglec10

In August 2019, a research paper published in *Nature* by Prof. *Weissman’s* team revealed another “don’t eat me” signal – CD24 [183]. CD24 is highly expressed on the surface of tumor cells and inhibits tumor cell phagocytosis by macrophages through binding to the inhibitory receptor Siglec10, which is highly expressed on the surface of macrophages. Siglec10, a member of the natural immune checkpoint, is a sialic acid binding Ig-like lectin 10, an inhibitory receptor recognized by sialic acid that regulates antibody production against sialylated antigens [184, 185]. In ovarian, triple-negative breast, and renal clear cell carcinomas, Siglec10 interacts with CD24 to inhibit immune cell activation and tumor cell phagocytosis. In studies of HCC, blockade of Siglec10^hi^ TAMs resulted in reduction of immunosuppressive molecule expression and enhancement of cytotoxic effects of CD8^+^ T cells, and also promoted the anti-tumor efficacy of PD-1 inhibitor, Pembrolizumab [186]. Siglec10 plays a critical tumorigenic role and its upregulation correlates negatively with the prognosis of patients. The inhibition of Siglec10 expression or therapeutic blockade of CD24 or genetic ablation has resulted in macrophage-dependent reduction in tumor growth and increased survival time in vivo [183, 185].

### Reprogramming of TAMs

TAMs include M1, which has anti-tumor effects, and M2, which expresses immunosuppressive and protumor factors; however, both are highly heterogeneous and plastic and can cross-regulate each other’s functions [187]. Stimulatory signals released by specific stimuli in the TME, such as tumor, immune, and stromal cells, can transform TAMs from one phenotype to another. For example, CXCL12 secreted by monocytes can repolarize M1-type TAMs to M2-type, making them key stromal cells in the TME that exert immunosuppressive functions and promote tumor progression and therapeutic resistance [188]. This provides research ideas for reprogramming TAMs to be anti-cancerous.

#### Chemotherapeutic drugs

Chemotherapeutic drugs can either selectively kill TAMs as one of their targets or regulate the response of macrophages to tumors by reprogramming the phenotype and function of TAMs based on their plasticity; thus, macrophages’ ability to present antigens and produce proinflammatory cytokines to stimulate cytotoxic T lymphocytes to fight tumor cells is restored. For example, cyclophosphamide enhances the production of proinflammatory cytokines (IL-12 and -6) and inhibits the secretion of protumor M2-related cytokines, including but not limited to IL-10. Cyclophosphamide-based immunotherapy for solid tumors has long been used in the clinic and several clinical trials are underway [90].

In a recent study, the combination of DNMTi 5-azacytidine (AZA) and α-diflomthylornithine (DFMO) was demonstrated to significantly improve the survival of patients with tumors and reduce the tumor load. A significant decrease in protumor M2 macrophages and increase in anti-tumor M1 macrophages in the TME suggested that this combined therapy can alter the polarization direction of macrophages in the TME, recruit M1 macrophages, and prolong survival time of patients [189].

#### TLR agonists

TLRs are important pathogen recognition receptors expressed by immune cells. A study demonstrated that TLR activation can reverse the function of TAMs [190]. In a tumor mouse model, the activation of the macrophage TLR signaling pathway upregulated the expression of M1-type specific markers, such as MHC-II and co-stimulatory molecules (e.g., CD86, CD80, and CD40), thereby enhancing the phagocytosis and anti-tumor activity of macrophages.

There are multiple TLR agonists available for the treatment of solid tumors, including imiquimod and 852A for TLR7, IMO-2055 for TLR9, and Rsiquimod (R848) for TLR7/TLR8 [43, 191]. There have been clinical trials for imiquimod in a variety of solid tumor types; and it also is the only FDA-approved topical treatment for squamous and basal cell carcinomas [192]. As for 852A, it has conducted six clinical trials in malignancies such as melanoma, HCC, and gynecologic malignancies.IMO-2055 was tested in clinical trials in solid tumors such as squamous cell carcinoma of the head and neck, NSCLC, renal cell carcinoma and CRC. Clinical trials in combination with IMO-2055 and erlotinib/bevacizumab for the treatment of progressive and chemotherapy resistance or advanced NSCLC showed a good tolerability and possible anti-tumor activity. [193]. IMO-2055 has shown in multiple clinical studies to be well tolerated and to be clinically active. This suggests that other anti-tumor agents might be combined with TLR9 agonists for further clinical trials [194]. According to studies in mice, administration of R848 could reprogram TAMs into M1 type and enhance the ADCP effect of TAMs, which in turn enhanced the therapeutic anti-tumor effects of TAMs [195]. Furthermore, BCG, a TLR2 and TLR4 agonist, is still used to treat patients with bladder cancer [196].

#### CD40 agonists

CD40, a member of the TNF receptor superfamily, is widely expressed on APC cells (including TAMs and DCs). CD40L, expressed on CD4^+^ T cells, is its ligand [197]. The activation of CD40/CD40L has been demonstrated to be able to upregulate MHC I molecule expression and produce proinflammatory factors (e.g., IL-12), in turn counteracting immunosuppression and initiating anti-tumor T cell immunity. Agonistic CD40 also promotes the conversion of TAMs into an anti-cancer phenotype via IFN-γ. The combination of anti-CD40 and anti-PD-1 significantly prolonged survival in mice with bladder cancer [198]. The binding of CD40 agonists to anti-CSF1R induces further reprogramming before the TAMs are depleted [43, 199]. Additionally, CCL5 produced by reprogrammed TAMs induces CD4^+^ T cell recruitment to the TME and remodels the TME to enhance anti-tumor immune responses [200].

Anti-CD40 induces anti-tumor immunity through multiple mechanisms, and several clinical trials are currently underway [198]. Simultaneously, the CD40 mAb, SHR-1704 has been approved for clinical use in the treatment of malignant tumors [201].

#### Tie2 inhibitors

Tie2 is a tyrosine kinase receptor for angiopoietin (Ang) 1, 2, and 4 and is mainly expressed in endothelial cells [202]. Ang/Tie2 kinase signaling pathway is a key angiogenic signaling axis in endothelial cells and is associated with recurrence and poor prognosis in cancer patients [203]. Tie2 is also expressed in pro-angiogenic macrophage (Tie2^+^ macrophage) subtypes and is involved in tumor vasculature and lymphangiogenesis, promoting cancer cell infiltration and metastasis [204]. Tie2^+^ macrophages also have an important role in tumor revascularization and recurrence after chemotherapy [205]. Hypoxic environment induces significant upregulation of Tie2 and its ligand Ang2 to promote tumor angiogenesis and maintain tumor growth [204]. In addition, resistance to bevacizumab and other VEGF-A pathway inhibitors in tumor patients is related to tumor infiltration owing to hypoxia and cell death after vascular regression by immune cells such as Tie2^+^ macrophages [206].

Tie2^+^ macrophages play a key role in tumor progression, which makes them ideal candidates for targeted therapy. The use of neutralizing antibodies against Tie2, siRNA targeting Tie2, and knockdown of Tie2 in hematopoietic stem cells all provide evidence of anti-tumor activity [207]. Rebastinib, an inhibitor that inhibits the Tie2 receptor on endothelial cells and macrophages, was shown to alter immune cells in ascites and increase the number of cytotoxic T cells in a preclinical model of ovarian cancer on the use alone and in combination with chemotherapy. Significantly, Rebastinib prolonged the survival of PDX and homozygous ID8 ovarian cancer mouse models in combination with chemotherapy [208]. In a xenograft mouse model, the MET/TIE2/VEGFR2 inhibitor altiratinib suppressed the growth and aggressiveness of glioblastoma. The combination of altiratinib with bevacizumab alleviated bevacizumab-induced hematological reconstitution, reduced infiltration of Tie2-expressing monocytes and upregulation of mesenchymal markers [209]. These results suggest that targeting Tie2^+^ macrophages in combination with other targeted therapies or chemotherapy has the potential to become a novel anti-tumor treatment strategy.

#### MARCO

As previously mentioned, expression of MARCO on TAMs indicates its anti-inflammatory pro-tumor subtype, and therefore, inhibition of MARCO is expected to remodel the phenotype of TAMs. In a mouse model of melanoma, targeting MARCO relieved the inhibitory effect of TAMs on NK cells, moreover, the combination of anti-MARCO antibody and PD-1/PD-L1 enhanced the efficacy of immune checkpoint therapy [98]. In NSCLC studies, inhibiting MARCO or blocking IL37 to suppress MARCO expression restores the anti-tumor activity of NK cells and T cells [96]. In prostate cancer studies, Marco blockade impaired lipid accumulation in TAMs, diminished TAMs aggregation at tumor sites, and increased MHC II expression in TAMs, thereby inhibiting tumor growth and metastasis. Anti-MARCO antibody also improves the anti-tumor effect of docetaxel in advanced prostate cancer [210]. Anti-MARCO treatment also limited the growth and metastasis of mouse breast cancer and melanoma, enhanced the immunogenicity of TME, and improved the therapeutic efficacy of anti-CTLA4 mAbs [211].

The available studies conclude that targeting MARCO is an effective approach to inhibiting TAMs' pro-tumor activity. Anti-MARCO antibodies inhibit tumor growth and metastasis, increase TME immunogenicity, and improve immune checkpoint therapy and chemotherapy effectiveness, making it a promising strategy that can be used in the treatment of solid tumors.

### CAR-M therapy

CAR-T therapies have made progress in the treatment of hematological malignancies but mostly failed when applied to the treatment of solid tumors. This is because the vascular network established by solid tumors “rejects” T cells, and even if T cells pass this barrier, various immune factors in the TME will attack them and weaken their ability to kill tumor cells. Therefore, in response to the bottleneck encountered by CAR-T cells, CAR-M cells have opened a unique path for the development of immunotherapy for solid tumors [212, 213].

In March 2020, Michael Klichinsky et al. published a study that demonstrated that the CAR gene manipulation of human macrophages can direct their phagocytic activity against tumors [212]. Chimeric adenoviral vectors can be used to engineer HER2 CAR-M macrophages. And transfection of viral vectors into PBMC cells derived from tumor-bearing patients to differentiate them into macrophages makes it more likely to maintain their sustained pro-inflammatory activity. This is a “two-in-one effect” that helps them to overcome the transformation of the tumor into an immunosuppressed state. The ability of macrophages to remain active lays the foundation for recognizing and engulfing cancer cells, enabling CAR-Ms to recognize, engulf, and kill tumor cells in the TME. One of the main advantages of CAR-M therapy is the ability to create a proinflammatory environment within the tumor. This proinflammatory state also makes the TME friendlier to other immune cells, such as T cells. Once inside the tumor, T cells can recognize tumor antigens presented by macrophages and target cancer cells for destruction.

A research published by *Jin Zhang*’s group at Zhejiang University in November 2020 clarified that CAR-expressing iPSC-derived macrophage (CAR-iMac) cells obtained from induced pluripotent stem cell (iPSC) differentiation was used for tumor immune cell therapy [213]. iPSCs are generated by reprogramming human PBMCs with the potential to differentiate into multiple somatic cells. They are derived from the patient and have the advantages of easy amplification, monoclonal gene modification, and ease of editing. The authors used the iPSCs to obtain CAR-iMac cells with not only a high yield (> 50 ×) and purity (nearly 100% CD11b/CD14 positive) but also gene expression profiles of macrophages and functions such as the phagocytosis and polarization of mature macrophages. When co-cultured with CD19^+^ lymphoma cells or mesothelin^+^ ovarian cancer cells, CAR-iMac cells exhibited antigen-dependent phagocytosis and killing of tumor cells; moreover, they exhibited the antigen-dependent secretion of proinflammatory cytokines as well as polarization toward M1-type macrophages. The cells have also demonstrated the ability to inhibit tumor cell growth in both hematological and solid tumor models in mice. glioblastoma.

In August 2022 Jiang et al. published their study deriving an intraluminal injectable nanopore-hydrogel superstructure for CAR-macrophage editing in vivo for post-operative immunotherapy of glioma [214]. In the glioblastoma multiforme (GBM) mouse model, nanopore-hydrogel introduces CAR genes targeting glioma stem cell (GSC) into the macrophage nucleus, resulting in the generation of GSC-specific CAR-Ms. CAR-Ms can specifically recognize and phagocytose GSCs and exert their antigen-presenting effects, which in turn stimulate adaptive anti-tumor immune responses and form immune memory [214]. In an orthotopic patient-derived glioblastoma humanized mouse model, macrophage-targeted editing nanocarriers (pCAR-NPs) and CD47 antibodies were co-delivered to the post-operative tumor cavity using a nanopore-hydrogel structure. This combination therapy synergistically enhanced the phagocytic efficacy of CAR-Ms on GSCs by targeting CD47 signaling, generating a strong anti-tumor immune response around the post-operative tumor cavity, which inhibited the recurrence of post-operative glioblastoma. [214]. This study shows that specific editing of CAR-Ms can inhibit the rapid recovery, proliferation and differentiation of residual GSCs after GBM surgery, which is expected to solve the problem of GBM recurrence caused by GSCs in the post-operative period.

CAR-M is an emerging and promising modality of operation. It has the following clear advantages in anti-solid tumor therapy [100]: ① CAR-Ms have the inherent tumor-homing ability of myeloid cells and are therefore able to enter solid tumors; ② CAR-Ms directly kill antigen-expressing tumor cells through phagocytosis or by secreting cytokines; ③ CAR-Ms promote an inflammatory environment by secreting cytokines and chemokines as well as by recruiting T cells and other leukocytes; ④ CAR-Ms can counteract the immunosuppressive microenvironment; and ⑤ CAR-Ms present antigens to T cells and induce adaptive immune responses.

In summary, CAR-M has demonstrated clear superiority in preclinical studies for the treatment of solid tumors. However, additional clinical trials are needed to validate its efficacy in the treatment of solid tumors. Currently, two clinical trials based on the CAR-M strategy have been approved by the FDA. The first is CT-0508, a drug candidate from CARISMA Therapeutics, through a phase I clinical trial using anti-HER2 CAR-M for patients with relapsed/refractory HER2-overexpressing tumors (NCT04660929). The other is MCY-M11 from Maxyte, which uses the mRNA transfection of PBMC to express CARs that targeting MSLN for the treatment of patients with recurrent/refractory ovarian cancer and peritoneal mesothelioma. Currently, volunteers are being recruited for a phase I clinical trial (NCT03608618).

## Conclusions and prospects

TAMs consist of tissue-resident macrophages and recruited monocyte-derived macrophages, which account for the largest proportion of the TME and play a critical role in tumor progression. In addition to affecting the TME, TAMs are also educated and modified in the TME to influence tumorigenesis, angiogenesis, the immune-suppressive microenvironment, tumor cell metastasis, and resistance to therapy through multiple mechanisms. Thus, identifying the regulatory mechanisms between TAMs and tumors, inhibiting their tumor-promoting effects, and enabling them to exert anti-tumor immune effects are currently hot research topics in the field of tumor immunotherapy. The strategies of reducing the number of infiltrating TAMs in the TME, reprogramming the phenotype and function of TAMs, inhibiting their protumor effects, and enhancing their anti-tumor phagocytic activity have yielded some progress in studies of immunotherapy for solid tumors. However, even the most successful anti-TAM therapies currently only benefit a small fraction of patients. Certain aspects of targeting macrophages should be paid more attention in future. (1) Recent studies have revealed the therapeutic value of targeted TAMs but also the complexity of their mechanisms of action at various stages of tumor progression. Therefore, in future studies, a more detailed classification of macrophage subtypes in TME and elucidation of the functions of various subtypes will provide a stronger basis for targeting TAMs for the treatment of solid tumors. (2) Furthermore, in targeting TAMs for solid tumors, researchers must systematically evaluate not only TAMs but also the patients themselves as a whole, so as to provide more accurate and effective treatment for more patients with solid tumors. (3) Clinically, the combination of targeting macrophage therapy into current cancer treatment should be considered to achieve better clinical outcomes. (4) CD47 mAb- or BsAb-based ADC drugs are also worth developing. (5) The preclinical results of CAR-M for other targets deserve further study, and in addition, the results of clinical trials related to CAR-M are also expected. (6) Side effects should also be taken into consideration. In conclusion, TAMs are reliable targets for tumor therapy. Targeting TAM is a promising strategy that will provide more precise and effective treatment options for solid tumor immunotherapy.

## Data Availability

Not applicable.

## Abbreviations

ADCC: Antibody-dependent cellular cytotoxicity
ADPC: Antibody-dependent cellular phagocytosis
CRC: Colorectal cancer
CSF-1R: Colony-stimulating factor 1 receptor
EMT: Epithelial-to-mesenchymal transition
GCs: Glucocorticoid
GM-CSF: Granulocyte–macrophage colony-stimulating factor
GRO: Growth-related oncogene
HCC: Hepatocellular carcinoma
HIF-1α: Hypoxia-inducible factor
HO-1: Heme oxygenase-1
IBC: Inflammatory breast cancer
ICIs: Immune checkpoint inhibitors
LPS: Bacterial lipopolysaccharides
MARCO: Collagen structural receptor
MDSCs: Bone marrow-derived suppressor cells
MMPs: Matrix metalloproteinases
NHPs: Nonhuman primates
NSCLC: Non-small-cell lung cancer
PBMC: Peripheral blood mononuclear cells
PDAC: Pancreatic ductal adenocarcinoma
SCLC: Small cell lung cancer
Siglec-10: Sialic-acid-binding Ig-like lectin 10
SIRPα: Signal regulatory protein α
SOCS: Suppressor of cytokine signaling
TAMs: Tumor-associated macrophages
TGF-β: Transforming growth factor-β
TLR4: Toll-like receptor 4
TME: Tumor microenvironment
TNF: Tumor necrosis factor
Tregs: Regulatory T cells
VEGF: Vascular endothelial growth factor
